# Interpretable machine learning for thermoelectric materials design with Kolmogorov–Arnold networks

**DOI:** 10.1038/s41598-026-44723-x

**Published:** 2026-03-19

**Authors:** Marco Fronzi, Michael J. Ford, Kamal Singh Nayal, Olexandr Isayev, Catherine Stampfl

**Affiliations:** 1 School of Physics, The University of Sydney, Camperdown, NSW 2006, Australia; 2 School of Mathematics and Physics, University of Technology Sydney, Haymarket, NSW 2007, Australia; 3 Department of Chemistry, Carnegie Mellon University, Pittsburgh, PA 15213, USA

**Keywords:** Engineering, Materials science, Mathematics and computing, Physics

## Abstract

The discovery of high-performance thermoelectric materials requires models that are both accurate and interpretable. Traditional machine learning approaches, while effective at property prediction, often act as black boxes and provide limited physical insight. In this work, we introduce Kolmogorov–Arnold Networks (KANs) for the prediction of thermoelectric properties, focusing on the Seebeck coefficient and band gap. Compared to multilayer perceptrons (MLPs), KANs achieve comparable predictive accuracy while offering explicit symbolic representations of structure-property relationships. This dual capability enables both reliable predictions and physically interpretable functional forms, providing insight into the governing mechanisms of thermoelectric behaviour. Benchmarking against literature baselines highlights their robustness and generalisability, demonstrating that KANs constitute a practical framework for reverse engineering materials with targeted thermoelectric performance and bridging the gap between predictive power and scientific interpretability.

## Introduction

Thermoelectric materials show significant promise for a variety of applications ranging from power generation to refrigeration^1^. Recent reviews have reaffirmed their potential for energy sustainability and waste heat recovery^2^. The efficiency of these materials is fundamentally tied to their Seebeck coefficient, electrical conductivity, and thermal conductivity^3^. However, the discovery and design of materials with efficient energy conversion remains a substantial challenge in the field^3–5^. A key metric used to assess thermoelectric performance is the dimensionless figure of merit, *zT*, defined as:

1 $$\begin{aligned} zT = \frac{S^2 \sigma T}{\kappa }, \end{aligned}$$

where *S* is the Seebeck coefficient, $\sigma$ the electrical conductivity, *T* the absolute temperature, and $\kappa$the total thermal conductivity^3^. The main difficulty lies in the complex interdependence between thermal and electrical conductivity, which cannot be easily decoupled to achieve a high *zT*^6,7^.

To understand these intricate relationships, quantum mechanical calculations –particularly those based on density functional theory (DFT) – can be employed^8,9^. DFT provides invaluable insights into the electronic structure and transport properties of materials, thereby guiding the design and discovery of new thermoelectric compounds^8,10,11^. However, such calculations are often computationally intensive and time-consuming, limiting their scalability for high-throughput screening^12^.

Machine learning (ML) has emerged as a transformative tool with the potential to revolutionise materials discovery^13^. When trained on existing data from quantum mechanical simulations and/or experimental results, ML models can rapidly predict the properties of novel materials, dramatically accelerating the pace compared to traditional computational methods^14^. Furthermore, these models can capture complex, non-linear patterns in data and generate accurate predictions across large datasets, making them particularly well-suited for exploring vast materials spaces^15^. However, a major limitation of most ML approaches is their reliance on correlation rather than causation, which often prevent the understanding of the underlying physical mechanisms^16,17^. As a result, their predictions, although highly valuable, typically serve as guidelines rather than definitive explanations in the materials discovery process^16–18^. These limitations motivate the exploration of interpretable machine learning architectures capable of capturing the underlying physics rather than merely correlating descriptors with target properties. Several recent efforts have attempted to reconcile predictive accuracy with physical interpretability by embedding stronger inductive biases into machine learning architectures. Recent advances in interpretable machine learning for materials science have pursued complementary strategies beyond descriptor-based regression. One direction focuses on learning physically grounded intermediate quantities–such as electronic Hamiltonians or density of states–and deriving target observables through established physical relations^19–21^. These approaches embed strong physical inductive biases and offer interpretability at the electronic-structure level.

A second strategy emphasises structurally transparent graph architectures, such as Capsule Graph Networks, which improve attribution at the node or motif level while maintaining predictive performance^22^.

In contrast, Kolmogorov-Arnold Networks (KANs) operate at the level of functional decomposition in descriptor space. Rather than reconstructing Hamiltonians or enforcing equivariance constraints, KANs approximate multivariate mappings as compositions of learnable univariate spline functions. This formulation yields analytic, differentiable surrogates directly linking structural embeddings to target properties.

We therefore position KANs as a complementary interpretable regression framework: suitable when high-quality structural embeddings are available and explicit structure-property mappings are desired. The mathematical foundation underlying this framework is the Kolmogorov–Arnold representation theorem, rooted in classical approximation theory.

The Kolmogorov-Arnold Network (KAN) represents a modern neural network design inspired by the Kolmogorov-Arnold representation theorem, which demonstrates that any multivariate continuous function can be expressed as a sum of one-dimensional functions of linear combinations of the inputs^18,23,24^. The KAN implements this concept through learnable univariate spline-based activation functions arranged in layers followed by summation, in contrast to the fixed nonlinearities used in traditional neural networks^23^. This design not only ensures universal approximation capability–allowing the representation of any continuous function–but also enforces local smoothness and continuity of the learned mapping, a property closely connected to approximation-theoretic analyses contrasting spline-based or structured shallow representations with deep piecewise-linear networks^24^. These properties later translate into enhanced stability and physical interpretability when modelling complex, correlated electronic systems. Kolmogorov-Arnold Networks allow better interpretability because each hidden unit functions as a one-dimensional relationship between a particular linear combination of input features and the target property. This unique architecture, coupled with learnable activation functions, provides a direct functional decomposition: the target property acquires an analytic functional form, which is highly useful for thermoelectric modelling due to the complex, coupled nature of the Seebeck coefficient and thermal conductivity. KANs provide explicit functional decomposition to disclose physical connections in thermoelectric materials, which conventional black-box models may hide, establishing an analytically sound method to model multivariable functions and enabling meaningful interpretation of predictions^25^.

While KANs are promising for capturing such physics-informed relationships, their performance must be contextualised against widely adopted black-box models to assess trade-offs in interpretability and accuracy.

In this study, we present an integrated machine learning framework for predicting the Seebeck coefficient–a key descriptor of thermoelectric performance–across a diverse set of bulk materials. To further evaluate the versatility of our approach beyond transport properties, we extend the modelling framework to predict the electronic band gap—an equally important but physically distinct property that governs many aspects of thermoelectric behaviour. Although not a direct transport coefficient, the band gap is a fundamental electronic property that strongly influences thermoelectric performance by affecting intrinsic carrier concentration, electrical conductivity, and bipolar conduction, particularly at elevated temperatures^26^. Its inclusion serves a dual purpose: first, as a complementary screening metric that reflects the quality of the underlying electronic structure; and second, as a benchmark for assessing the flexibility and generalisability of the KAN architecture. Unlike the Seebeck coefficient, which is highly sensitive to the curvature and asymmetry of the bands near the Fermi level^27,28^, the band gap is governed by broader features of the electronic structure. Strong performance across both properties demonstrates the model’s robustness in capturing physical trends that span from global electronic characteristics to fine-grained transport behaviour.

To provide a rigorous benchmark, we also train a multi-layer perceptron (MLP) on the same dataset, enabling direct comparison between the interpretability-accuracy trade-offs of KAN and a conventional deep learning architecture.

## Methodology

The methodological framework of this study combines conventional machine learning baselines with novel interpretable neural architectures to predict key electronic and thermoelectric properties of crystalline materials. Our objective is twofold: first, to establish a reliable reference using well-understood models, and second, to assess the performance and interpretability gains enabled by Kolmogorov–Arnold Networks. To this end, we employed multilayer perceptrons (MLPs) as benchmark models, providing a standard against which KAN results can be rigorously compared.

### Multilayer perceptrons as benchmark models

**Fig. 1:**
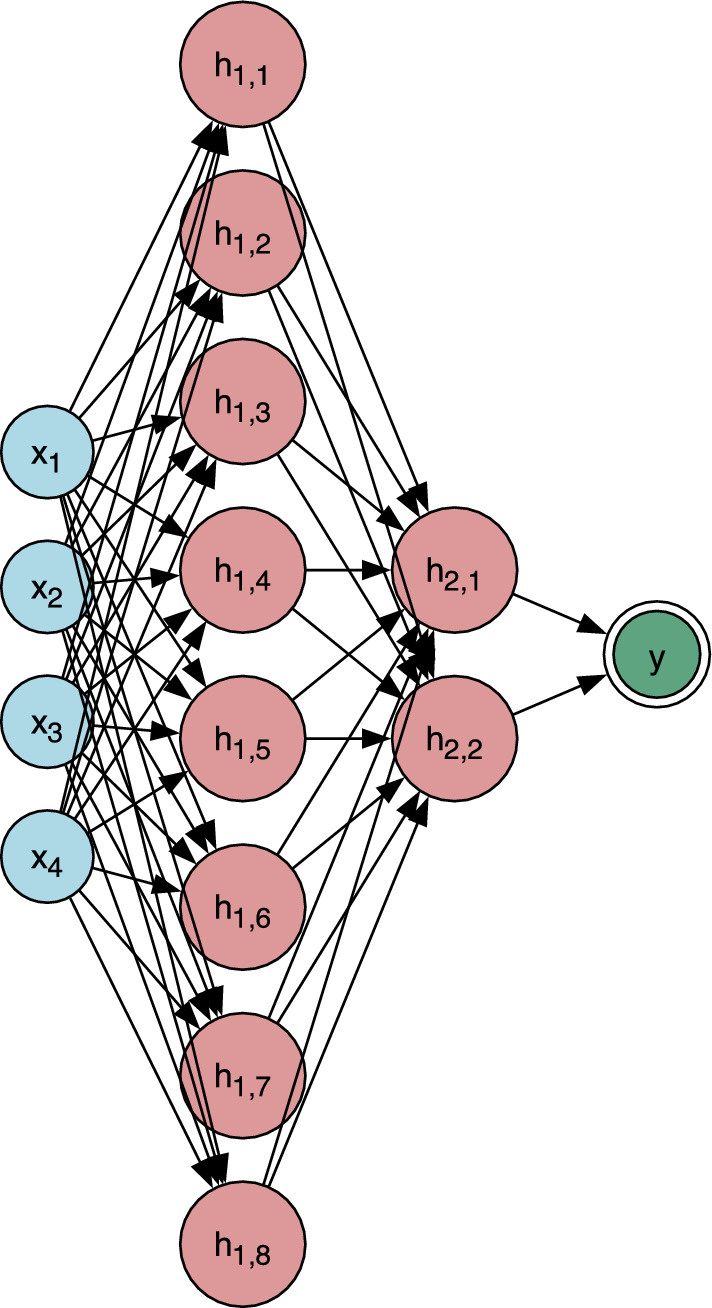
Schematic of a standard feedforward neural network. Each hidden node computes a weighted sum of its inputs, applies a non-linear activation function, and propagates the signal forward. The output is a scalar regression target, such as the Seebeck coefficient or band gap.

An MLP is a fully connected feedforward neural network in which nodes are arranged in successive layers, as shown in Fig 1. Each neuron computes a weighted sum of its inputs followed by a nonlinear activation:

2 $$\begin{aligned} f(x_1, \ldots , x_n) = \sigma \left( \sum _{i=1}^{n} w_i x_i + b \right) , \end{aligned}$$

where $w_i$ and $b$ denote the learnable weight and bias parameters, and $\sigma$ represents the activation function. In this work, rectified linear units (ReLU) were chosen for the hidden layers owing to their computational efficiency and ability to mitigate vanishing gradients, while a linear activation was adopted in the output layer to accommodate regression tasks such as band gap and Seebeck coefficient prediction.

The models were implemented in the PyTorch framework and trained on datasets split into training (80%) and test (20%) subsets, with performance further validated using 5-fold cross-validation, and early stopping was triggered with a patience of 80 epochs based on validation loss.

Hyperparameters were selected through systematic grid search across layer sizes, learning rates, and patience values. The Adam optimiser was employed with a fixed learning rate of $10^{-3}$, weight decay of $10^{-4}$, and default momentum parameters $(\beta _1 = 0.9, \beta _2 = 0.999)$^29^. Training minimised the mean squared error (MSE) loss function and proceeded for up to 2000 epochs. Model selection was guided by multiple metrics, including the coefficient of determination ($R^2$), root mean squared error (RMSE), and mean absolute error (MAE), ensuring accurate predictions with minimal train–test degradation.

The optimal architecture identified through this process was $[128, 128, 64, 1]$. This configuration consistently achieved high $R^2$ values alongside low RMSE and MAE across validation folds. Importantly, the small discrepancies between training and test metrics highlighted the strong generalisation ability of the model, validating its role as a robust benchmark against which KAN performance could be assessed.

### Kolmogorov–Arnold networks

**Fig. 2:**
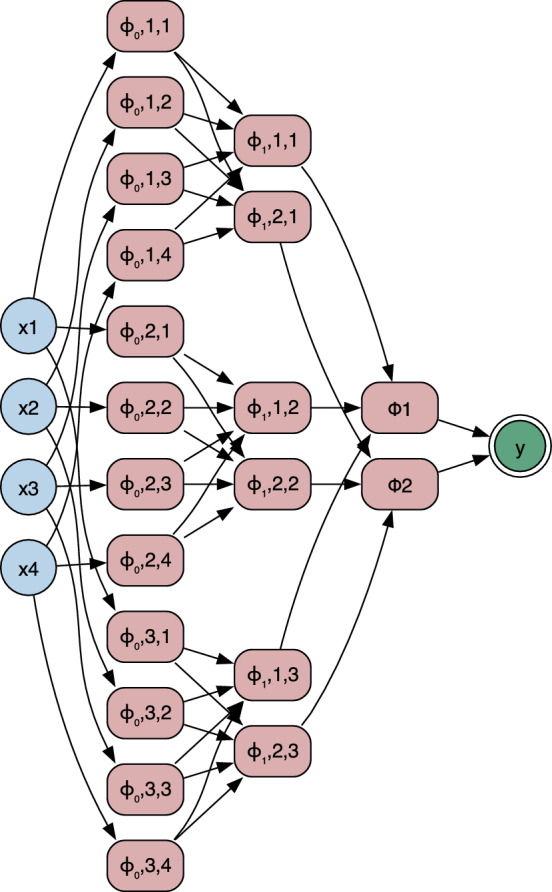
Schematic representation of a Kolmogorov–Arnold Network. Each input $x_i$ is transformed by a set of learnable univariate functions $\phi _{q,p}(x_p)$, summed, and then mapped by outer functions $\Phi _q$ to produce the final output *y*. This architecture directly embodies the compositional structure dictated by the Kolmogorov–Arnold representation.

Kolmogorov–Arnold Networks are a neural architecture, schematically shown in Fig. 2derived from the Kolmogorov–Arnold representation theorem, which guarantees that any multivariate continuous function can be expressed as a finite superposition of univariate continuous functions combined with binary addition operations^30–32^. Formally, the representation is written as:

3 $$\begin{aligned} f(x_1, \ldots , x_n) = \sum _{q=0}^{2n} \Phi _q \left( \sum _{p=1}^{n} \phi _{q,p}(x_p) \right) , \end{aligned}$$

The index *q* runs from 0 to 2*n*, ensuring a finite set of superpositions. In this representation, $\phi _{q,p}$ denotes the inner univariate function acting on input $x_p$, and $\Phi _q$ denotes the outer univariate function applied to the aggregated contributions from all inputs. This decomposition establishes the theoretical foundation for universal approximation when implemented with shallow, fixed-width networks, which require at most $2n+1$hidden nodes^30^.

Unlike multilayer perceptrons (MLPs), which place fixed nonlinear activations at nodes and linear transformations on edges, KANs invert this design:

- **Nodes** act as summation units.
- **Edges** implement learnable nonlinearities parameterised as one-dimensional spline functions.

This edge-based parametrisation provides intrinsic interpretability, as each connection corresponds to an explicit functional transformation of its input. This directly encodes functional transformations along each network edge, enabling symbolic post-processing of the trained network as described below. We reconfigure the model to evaluate symbolic representation, where the output of each neuron in a KAN layer can be expressed as:

4 $$\begin{aligned} y_i = \sum _{j} w_{ij} \, \phi _{ij}(x_j), \end{aligned}$$

where $x_j$ denotes the *j*-th input feature, $\phi _{ij}(\cdot )$ is a trainable univariate function associated with the connection from input *j* to neuron *i*, and $w_{ij}$ is a scalar weight. This formulation can be seen as the practical, layer–wise realisation of the Kolmogorov–Arnold functional decomposition shown in Equation (3), in which each multivariate function is represented as a finite sum of outer functions $\Phi _q$ applied to inner sums of univariate transforms $\phi _{q,p}$. In the network implementation, the inner sum over *p* corresponds to the aggregation $\sum _j w_{ij} \, \phi _{ij}(x_j)$, while the outer function $\Phi _q$ is either absorbed into the next layer or represented by subsequent $\phi$ transformations. This mapping bridges the theorem-level representation and the computational architecture, preserving the universal approximation property while enabling gradient-based optimisation.

Here, KANs were implemented using the open-source PyTorch and pykanlibraries, which provide full hyperparameter control and differentiable optimisation of spline-based activations^31,33^. Each spline was initialised on a fixed grid and parameterised by cubic B-splines ($k=3$), with grid resolution $G=12$. The learning objective combined standard prediction error minimisation with $\ell _1$-type sparsity regularisation, promoting compact models.

The network architecture consisted of an input layer matching the feature dimension (128), one hidden layer (width 16), and a single output node. Training employed the Adam optimiser in the initial phase, whereas the final model refinement was performed using the limited-memory Broyden–Fletcher–Goldfarb–Shanno (LBFGS) optimiser. Learning rate and weight decay were set to $10^{-3}$ and $10^{-4}$, respectively, for up to 2000 epochs, with early stopping (patience of 80 epochs) based on validation MSE. Two additional regularisation terms were incorporated: an $\ell _1$ sparsity penalty ($\lambda = 0.01$) to promote compactness, and an entropy-based smoothness penalty ($\lambda _{\text {entropy}} = 0.1{-}0.2$) to stabilise functional representations. The regularisation term is weighted by $\lambda _{\text {entropy}}$, which controls the smoothness of the learned spline functions. In the Gaussian Mixture Model (GMM) used for output scaling, $\alpha$ is the mixing coefficient determining the relative contribution of each Gaussian component, and $p_\textrm{GMM}$ represents the corresponding membership probability.

#### Symbolic extraction and interpretability

After the numerical optimisation, symbolic post-processing was performed to reconstruct analytic surrogates for the most influential spline functions. Models clarity was improved by pruning weak or non-contributing edges, identified by coefficient magnitudes below a fixed threshold, set to 0.01. Removing these edges reduced complexity and eliminated noisy contributions, yielding more compact and physically meaningful symbolic expressions.

**Fig. 3:**
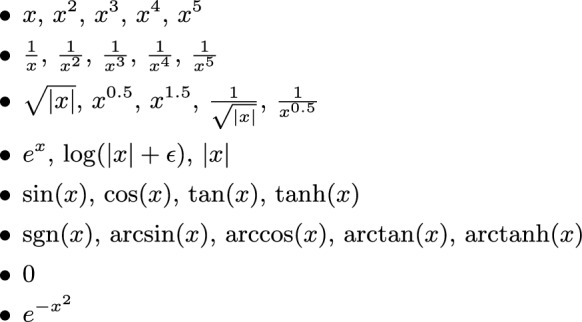
Set of elementary functions used as basis candidates ($\mathcal {D}$). Here, $\epsilon$ is a small positive constant (e.g., $10^{-6}$) added to avoid singularities in logarithmic and reciprocal functions.

The symbolic form of each learned activation was then extracted by fitting candidate functions from a predefined library ($\mathcal {D}$), which in its final form included a broad set of elementary functions (Fig. 3).

Each active univariate spline $\phi _{ij}(x)$ is approximated with a compact symbolic surrogate $\tilde{\phi }_{ij}(x)$ drawn from a dictionary $\mathcal {D}$. The chosen elementary functions constitute a general-purpose approximation dictionary rather than a physics-imposed basis. The inclusion of trigonometric, rational, and saturating nonlinearities reflects functional behaviours frequently encountered in electronic-structure and transport modelling (e.g., oscillatory dispersion effects, inverse scaling relations, and saturation-like responses). However, no explicit physical functional form was enforced during training. Symbolic expressions were selected purely on the basis of data fitting within this predefined dictionary. This broad function set enables representation of polynomial and non-polynomial relationships, oscillatory structure, and asymptotic behaviour, while preserving interpretability without introducing hard-coded physical constraints. Candidate expressions are fitted by least squares on the knot grid and validated on held-out points sampled within the empirical support of *x*. The reported complexity score $c$ in the tables is a discrete proxy for interpretability:

- $c=1$: affine or single low-order polynomial (*x*, $x^2$).
- $c=2$: single-elementary nonlinearity with bounded range or simple rational form (e.g., $\sin$, $\cos$, 1/*x*) with one affine phase/scale.
- $c=3$: higher-curvature or singular forms (e.g., $\tan$) or shallow compositions of two primitives (e.g., $\sin (ax+\beta )+\gamma x$).
- $c\ge 4$: piecewise or multi-term compositions (not used when a lower-*c* surrogate attains $r^2\ge 0.996$).

To extract symbolic relations from the trained models, we employed an $R^2$ acceptance threshold for symbolic regression. We set the cutoff value to $R^2 = 0.9$ provided the best trade-off between interpretability and reliability. At this threshold, the symbolic functions retained sufficient accuracy to capture the dominant structure–property relationships, while still allowing the inclusion of approximate functional forms that may reflect underlying physical trends.

The extracted analytical expressions facilitated the mapping of learned relationships back to physically interpretable descriptors, enabling direct comparison with known theoretical forms and empirical trends.

### Baseline regression models

To ensure a rigorous and fair comparison, we benchmarked seven widely used nonlinear tabular regression models: Random Forest, XGBoost, LightGBM, CatBoost, Support Vector Regression (SVR), Kernel Ridge Regression, and TabPFN-2.5. All models were trained using the same 128-dimensional CrystalFormer embeddings and identical train/test splits to eliminate representation- or split-induced bias.

Hyperparameter optimisation was performed independently for each model using five-fold cross-validated random search with 25 sampled configurations per model. The best-performing configuration was selected based on the lowest mean validation RMSE across folds. Random seeds were fixed to ensure reproducibility, and preprocessing strategies (including feature scaling and target transformations where applicable) were applied consistently across models.

For TabPFN-2.5, the default regression checkpoint was employed following the standard inference protocol recommended by the developers. As TabPFN operates via in-context learning using a pre-trained foundation model, no dataset-specific gradient-based fine-tuning was performed.

Comprehensive implementation details, including search spaces, optimisation budgets, early stopping strategies, and preprocessing procedures, are provided in the Supplementary Information.

### Dataset and target properties

The dataset used in this study is derived from the ricci_boltztrap_mp_dataset, which contains thermoelectric and electronic properties computed via DFT followed by semi-classical Boltzmann transport analysis using the BoltzTraP code^34–36^. The crystal structures originate from the Materials Project database, ensuring consistent treatment of exchange–correlation effects and structural optimisation parameters across the dataset^37^.

The target properties include the electronic band gap and the Seebeck coefficients for electrons ($S_n$). Band gaps were obtained from standard DFT calculations and subsequently corrected to improve alignment with experimental trends. The Seebeck coefficients were calculated under the constant relaxation time approximation by solving the linearised Boltzmann transport equation, using a fixed temperature of 300 K and a chemical potential aligned with the intrinsic Fermi level. Units are $\mu \mathrm {V/K}$ and $S_n$, and electronvolts (eV) for the band gap. Although widely used in materials informatics, these computed quantities carry known systematic limitations that are discussed in the Discussion section. Despite these factors, the relative trends and rank ordering of materials are generally preserved, and such limitations are negligible in the present work, as the primary objective is the evaluation of KAN architectures for predicting complex target properties and interpreting the learned structure–property relationships, assessing the utility of the models for reverse engineering purposes.

**Fig. 4:**
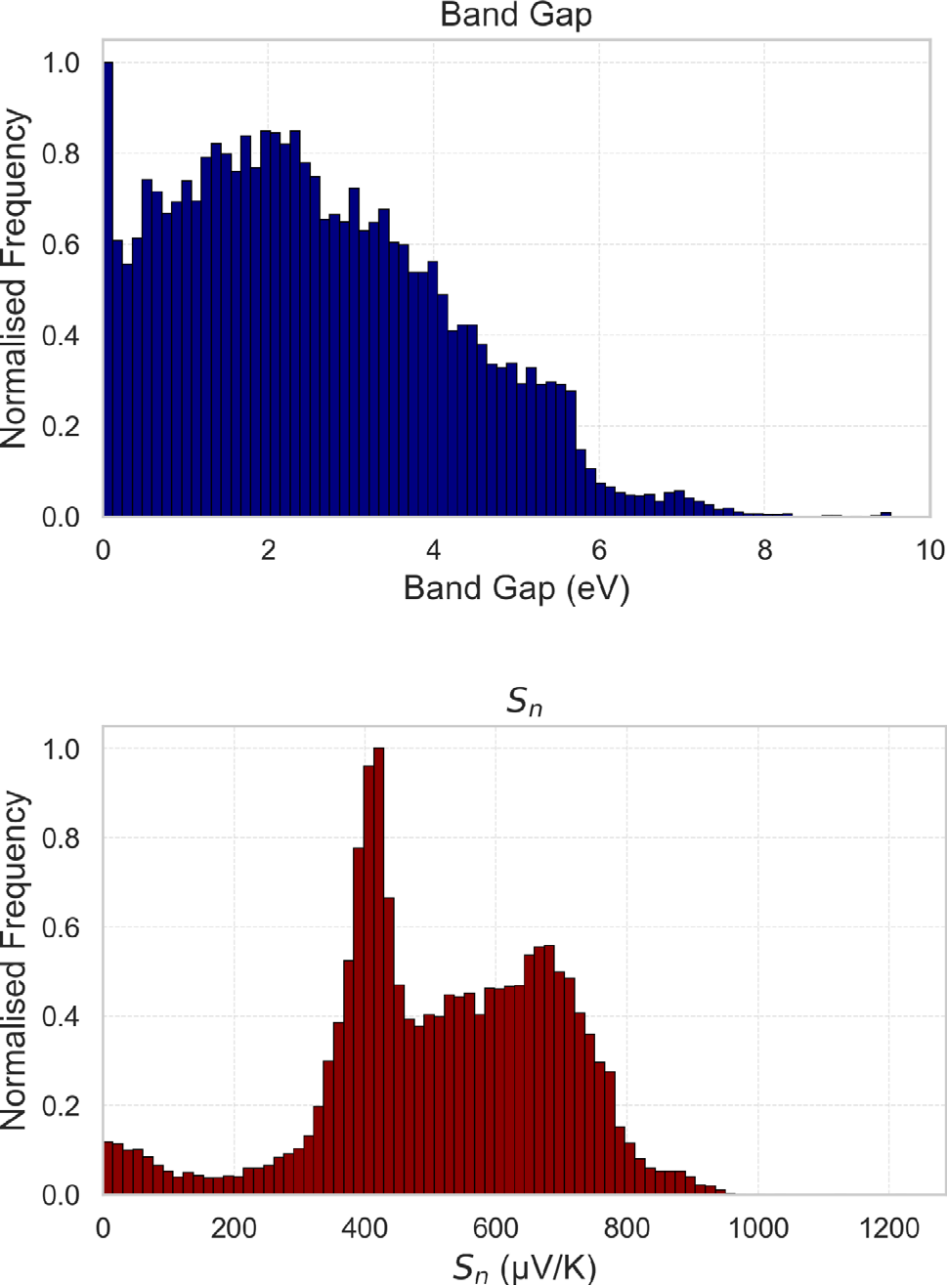
Histograms showing the distribution of key target properties in the dataset. **Top:** Band gap, which is skewed right with a large number of materials having small band gaps and a long tail extending beyond 6 eV. **Bottom:** Seebeck coefficient for electrons ($S_n$), displaying a bimodal distribution with peaks near 400 $\mu$V/K and 650 $\mu$V/K.

The statistical distribution of the target properties is shown in Fig. 4. The band gap distribution is right-skewed, with a large proportion of materials exhibiting small band gaps and a long tail extending beyond 6 eV, whereas the electron Seebeck coefficient exhibits a left-skewed bimodal distribution with peaks near 400 $\mu$V/K and 650 $\mu$V/K.

### Crystalformer representations and feature encoding

To describe the crystal structure space effectively, we use *Crystalformer*, a novel Transformer framework for periodic structure encoding. Crystalformer is a Transformer-based encoder inspired by Graphormer, which applies fully connected attention between atoms in a molecule^38,39^. To capture both local atomic environments and long-range interactions in particular, it extends this approach to introduce infinitely connected attention arising from the periodicity in crystals, formulated as an infinite summation of interatomic potentials in an abstract feature space. The Crystalformer architecture follows the Transformer^40^encoder design with stacked self-attention blocks made of two residual connections linking a multi-head attention layer and a shallow feed-forward network, but unlike the original model it removes Layer Normalization entirely to help stabilize training^40^. It builds upon the success of materials graph networks while incorporating positional (both spatial and edge) encoding for periodicity-aware modeling and ensuring permutation, SE(3) and periodic invariance (both supercell and periodic-boundary shift).

The use of Crystalformer embeddings provides several advantages: (i) it captures high-order geometric correlations and structural motifs not easily represented by classical hand-crafted features; (ii) it enables the transfer of knowledge from large crystal datasets to our thermoelectric prediction task; and (iii) it supports end-to-end differentiability and integration with downstream prediction models. The physics-inspired treatment of infinitely connected attention leads to learned structural embeddings, which enhance both the predictive accuracy and robustness of the models trained on them.

In this work, Crystalformer was used as a *supervised featuriser*that transforms input crystal structures into fixed-length, continuous vector embeddings. Each structure, initially represented by its atomic positions, species, and lattice parameters, was encoded into a learned structural representation by training on a large dataset of materials from the Materials Project and the Open Quantum Materials Database^37,41^. The encoder transforms atom embeddings into a latent structural representation, which is then aggregated via global average pooling into a single 128-dimensional vector per material.

To integrate these latent representations into our predictive pipeline, we extracted the final vector embeddings from the penultimate layer of the Crystalformer encoder, resulting in a 128-dimensional vector for each input material. Prior to model training, all features were standardised to zero mean and unit variance using the training set statistics. Crystalformer code and training settings were directly adapted from the original paper and modified to account for multi-target training, as well as to save the final layer representation before the regression layer for use as the latent representation of the input crystal structures. All training data was used to train the Crystalformer model with a batch size of 256 materials for 250 epochs.

#### Rationale

These transformations were designed to preserve the physical interpretability of the target variables while improving their suitability for learning with smooth, spline-based architectures. By addressing skewness, heavy tails, and multimodality, the preprocessing pipeline enhances both model convergence and predictive stability.

## Results

### Data preprocessing and target normalisation

#### Feature scaling

The input feature matrix $\textbf{X}$, comprising structural and compositional descriptors extracted from learned embeddings, was standardised to zero mean and unit variance using StandardScaler:

5 $$\begin{aligned} \textbf{X}_{\text {scaled}} = \frac{\textbf{X} - \mu }{\sigma }, \end{aligned}$$

where $\mu$ and $\sigma$ denote the column-wise mean and standard deviation, respectively. Standardisation mitigates internal covariate shift and accelerates convergence in neural architectures such as MLPs and KANs.

#### Target scaling

Because the target variables exhibited markedly different statistical distributions, property-specific normalisation procedures were applied to improve numerical stability and regression accuracy. Each property—the electronic band gap and the electron Seebeck coefficient ($S_n$)—was modelled independently. Preprocessing was carried out in Python using the scikit-learn library, and the fitted scalers were stored with joblib to guarantee consistent transformations during training and inference. The normalisation was designed to approximate Gaussian-like target distributions, a choice that facilitates convergence and stabilises optimisation in gradient-based learning algorithms.

*a. Band gap.* The DFT-predicted band gaps were approximately unimodal and symmetric; thus, a standard $z$-score transformation using StandardScaler was applied:

6 $$\begin{aligned} y_{\text {scaled}}^{(\text {band\_gap})} = \frac{y - \bar{y}}{\textrm{std}(y)}. \end{aligned}$$

*b. Electron Seebeck coefficient* ($S_n$).

For the Seebeck coefficients, strongly non-Gaussian, bimodal and strongly skewed distributions required composite normalisation procedures.

To address this, a multi-step transformation was employed: (i) log-sign transformation to suppress heavy tails:

7 $$\begin{aligned} y' = \text {sgn}(y) \cdot \log (1 + |y|), \end{aligned}$$

(ii) followed by Gaussian Mixture Model (GMM) normalization to approximate a Gaussian-like target distribution suitable for gradient-based optimization.

8 $$\begin{aligned} y_{\text {scaled}}^{(S_n)} = \mathrm {GMM\_Scaler}(y'), \end{aligned}$$

where $\mathrm {GMM\_Scaler}$ is a composite transformation using the learned parameters of the Gaussian components, a necessity for robustly modeling the **bimodal distribution** observed for the electron Seebeck coefficient.

### Multilayer perceptron network baseline performance

**Fig. 5:**
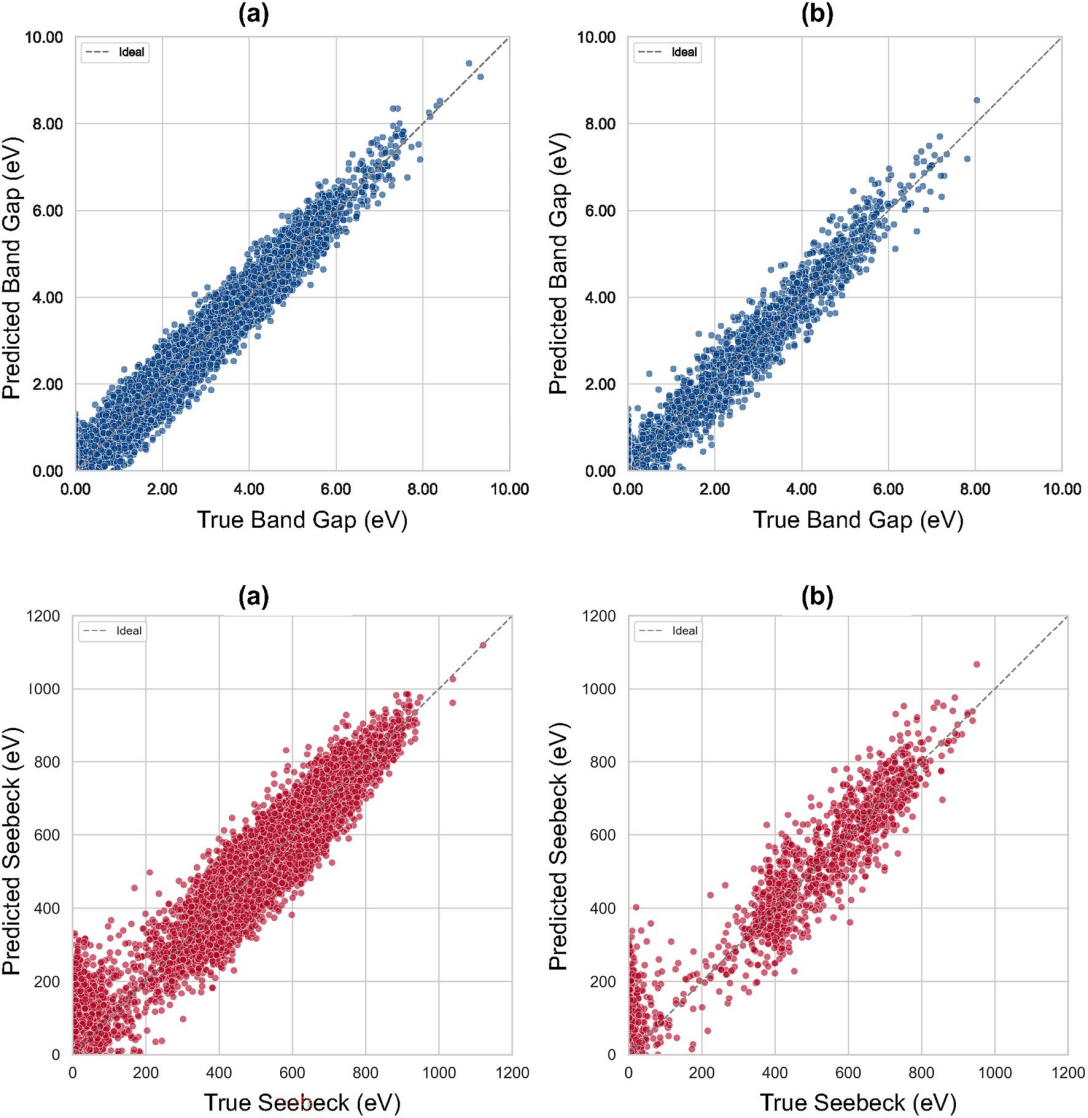
Parity plots for predicted vs. true values of the band gap and $S_n$ (top and bottom, respectively) using the MLP model. Each property is shown for both training and test sets (left to right). The diagonal line represents perfect prediction.

We implemented a fully connected neural network MLP baseline with architecture [128, 128, 64, 1], selected via grid search on CrystalFormer embeddings using $R^2$ and MSE as reference metrics, and trained it to predict the band gap and Seebeck coefficient. The filtered dataset includes 15,000 structures split 80/20 into training and test sets. Figure 5 presents parity plots for train and test partitions, while Table 1 reports the corresponding metrics.

For band gaps, the MLP reach high fidelity with $R^2=0.956$ and sub-0.1 eV median absolute errors (MAE $=0.087$ eV; Table 1). Parity scatter in Fig. 5 is tightly clustered along the diagonal, and the modest train-test gap ($R^2_\textrm{train}=0.982$ vs. $R^2_\textrm{test}=0.956$) indicates controlled variance and good generalisation. For $S_n$, the model preserves strong rank order ($R^2\textrm{test}=0.895$ reported in Table 1) with RMSE values of 73.6. The slightly broader residual spread visible in Fig. 5 for small |*S*| is consistent with the known heterogeneity and skew/bimodality of Seebeck distributions in chemically diverse sets.

The band gap accuracy compares favourably with classic tree ensembles and earlier deep models: our MAE and RMSE are on par with, or better than, LightGBM/Random Forest baselines on related datasets and markedly better than earlier MLPs on 2D sets (RMSE $\sim$0.47 eV); see Table 4for context^42,43^. For Seebeck coefficients, our absolute errors (MAE $\approx 39$–$46~\mu$V/K; Table 1) are slightly higher than the best-in-class boosted ensembles and specialised deep nets reporting $\sim$20–$37~\mu$V/K^44–47^. However, those studies often target narrower chemistries or employ tailored feature engineering, whereas our single MLP–trained on a broad, mixed set using a unified representation–reaches consistent performance across both band gap and $S_n$ with limited tuning (Table 1, Fig. 5). This makes it a robust and reproducible baseline for subsequent interpretability–focused models.

The architecture used here offers a compact parameterisation on the order of $\approx 2.4\times 10^4$ parameters (Table 2) that captures the relatively smooth structure–property relation for band gaps, while being slightly less suited describe nonlinearities that govern the Seebeck response. The small train–test deltas across all targets (Table 1) suggest neither severe underfitting nor memorisation; instead, the residual errors for $S_n$ likely reflect physics not directly encoded in the features (e.g., carrier concentration), rather than deficiencies in optimisation.

As a baseline, the MLP coupled to CrystalFormer embeddings delivers (i) state-of-the-art band-gap accuracy relative to general-purpose baselines and (ii) competitive, stable Seebeck predictions on a chemically diverse dataset. This establishes a reliable reference for the KAN models assessed in the next section.

**Table 1:** Performance metrics (train and test sets) for MLP models trained with CrystalFormer features on the filtered 15,000-sample dataset.

Property	Set	R$^2$	MSE	RMSE	MAE
Band Gap	Train	0.982	0.0113	0.106	0.065
Band Gap	Test	0.956	0.0225	0.150	0.087
$S_n$	Train	0.909	4512.4	67.17	34.44
$S_n$	Test	0.895	5473.7	73.62	38.53

**Table 2:** Parameter breakdown for a conventional MLP and a KAN. For the MLP, parameters are separated into edge weights and biases per layer. For the KAN, the decomposition shows the actual trainable parameter classes reported by PyKAN.

Model	Component	Count
**MLP (128–128–64–1)**		
	Edges (In$\rightarrow$128)	16,384
	Bias (128)	128
	Edges (128$\rightarrow$64)	8,192
	Bias (64)	64
	Edges (64$\rightarrow$1)	64
	Bias (1)	1
	**Total**	**24,833**
**KAN (128–16–1)**		
	Spline coefficients	30,960
	Grid/knots	2,736
	Affine parameters	12,418
	Bias	34
	Other	4,128
	**Total**	**50,276**

### Kolmogorov–Arnold networks for descriptor discovery and reverse engineering

After performing a grid search with CrystalFormer descriptors (using $R^2$ and MSE as reference metrics), we selected KAN architectures of [128, 16, 1] across the three targets. As summarised in Table 2, our KANs involve more parameters per connection, though by reducing the number of hidden units and layers their overall size can be kept comparable to that of MLPs.

Despite this comparable parameter count, training KANs is markedly slower. In MLPs, forward and backward propagation reduce to highly optimised matrix multiplications and outer products, operations that can scale efficiently on GPUs. KANs, however, demand the evaluation of spline basis functions and their derivatives for each edge. With $G{+}k=15$ coefficients per connection ($G=12$ grid points, $k=3$ spline order), every pass requires costly polynomial interpolation and gradient calculations. Furthermore, continuity constraints couple neighbouring spline coefficients, complicating optimisation and increasing memory overhead by storing spline activations and their derivatives. Together, these factors explain why KAN training is computationally more demanding, even when the number of parameters is comparable to MLPs.

On a multi-core CPU with optimised libraries for dense linear algebra, we observed significant differences: a baseline MLP with $\sim$24k parameters converged within minutes, whereas a KAN of comparable size ($\sim$50k parameters, cubic splines with $G{+}k=15$) required on the order of eight hours. In effect, the per-parameter cost of KAN training was over two orders of magnitude higher, reflecting the computational burden of spline evaluations and their coupled optimisation. We alleviated this overhead by pruning redundant connections and adopting smaller initial grids, which reduced training time without compromising predictive accuracy.

**Fig. 6:**
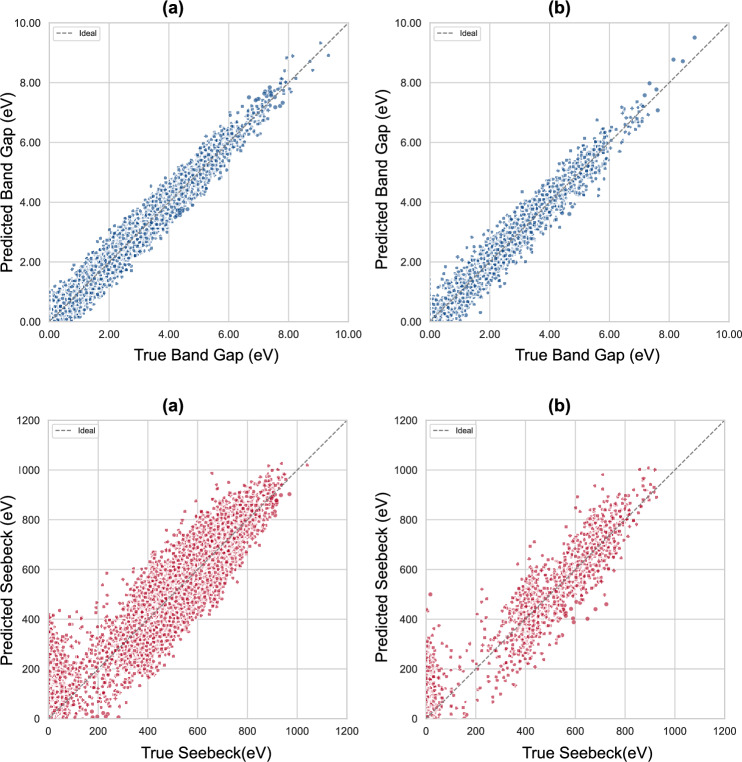
Parity plots for predicted vs. true values of the band gap and $S_n$ (top and bottom, respectively) using the KAN model. Each property is shown for both training and test sets (left to right). The diagonal line represents perfect prediction.

The trained KAN models achieved consistently high predictive performance across band gap and Seebeck coefficients, with parity plots (Fig. 6) showing strong agreement between predicted and true values in both training and test sets. The performance metrics are summarised in Table 3, demonstrating that KANs performance is comparable to multilayer perceptrons (Table 1), albeit with the added benefit of interpretability. In particular, KAN achieves higher R$^2$ for band gap (0.968 vs 0.956) with comparable RMSE, though at higher MSE, yet remain competitive with recent state-of-the-art machine learning approaches reported in the literature (see Table 4), including gradient-boosted decision trees, deep neural networks, and graph-based models. These results establish KANs as a viable alternative to conventional architectures.

**Table 3:** Performance metrics (train and test sets) for KAN models trained with CrystalFormer features on the filtered 15,000-sample dataset.

Property	Set	R$^2$	MSE	RMSE	MAE
Band Gap	Train	0.974	0.0225	0.112	0.072
Band Gap	Test	0.968	0.0324	0.146	0.094
$S_n$	Train	0.895	5260.7	71.27	35.12
$S_n$	Test	0.851	7085.1	84.17	39.09

**Table 4:** Representative machine learning models reported between 2015 and 2025 for predicting band gaps and Seebeck coefficients of inorganic materials. Results include both recent models and selected high-performing baselines from earlier studies.

Target property	Model type	$R^2$	MSE	RMSE	MAE
Band gap (2D materials)	GBDT^43^	0.92	–	0.24 eV	–
Band gap (2D materials)	MLP^43^	0.70	–	0.47 eV	–
Band gap (perovskites)	LightGBM^42^	0.934	–	–	0.302 eV
Band gap (perovskites)	XGBoost^42^	0.911	–	–	0.350 eV
Band gap (perovskites)	Random Forest^42^	0.921	–	–	0.320 eV
Band gap (perovskite oxides)	Ensemble model^48^	0.86	$\sim$0.07 eV$^2$	$\sim$0.26 eV	0.18 eV
Band gap	SVR/GBDT + SISSO^49^	–	–	0.36 eV	–
Band gap	CGCNN (domain adaptation)^50^	–	–	–	0.23 eV
Band gap (mixed materials)	CrystalFormer^51^	0.97	–	0.048 eV	0.033 eV
Band gap (semiconductors)	GNN + spectral features^52^	0.945	–	0.11 eV	0.08 eV
Band gap (inorganics)	Deep KRR + SOAP^53^	0.89	–	–	0.22 eV
Seebeck ($S_n$/$S_p$)	CraTENet^46^	0.78	–	–	$\sim$114 $\mu$V/K
Seebeck ($S_n$/$S_p$)	RF^46^	0.79	–	–	$\sim$141 $\mu$V/K
Seebeck ($S_n$/$S_p$)	CraTENet+gap^46^	0.96	–	–	$\sim$49 $\mu$V/K
Seebeck ($S_n$/$S_p$)	RF+gap^46^	0.96	–	–	$\sim$54 $\mu$V/K
Seebeck ($S_n$/$S_p$)	NN + elemental features^47^	0.96	–	–	31–39 $\mu$V/K
Seebeck ($S_p$)	GBT/CatBoost^44^	0.73	–	85	55 $\mu$V/K
Seebeck ($S_p$, half-Heusler)	XGBoost ensemble^45^	0.95	–	–	20.8 $\mu$V/K
Seebeck ($S_n$, half-Heusler)	LightGBM ensemble^45^	0.94	–	–	20.8–37.0 $\mu$V/K
Seebeck (mixed, exp. data)$^{\dagger }$	XGBoost^54^	0.90	–	–	21.1 $\mu$V/K
Seebeck (inorganics)	Matminer baseline^55^	0.85	–	–	36.70 $\mu$V/K
Seebeck (inorganics)	GNN (symmetry-aware)^52^	0.93	–	–	19–21.7 $\mu$V/K

$^{\dagger }$ Trained on a broad experimental thermoelectric dataset of 5,205 samples; model achieved $R^2 \ge 0.90$ for multiple transport properties.

**Note:** “–” indicates that the metric was not explicitly reported. Errors are in eV for band gap and in $\mu$V/K for the Seebeck coefficient.

### Robustness and generalization across chemical space

**Fig. 7:**
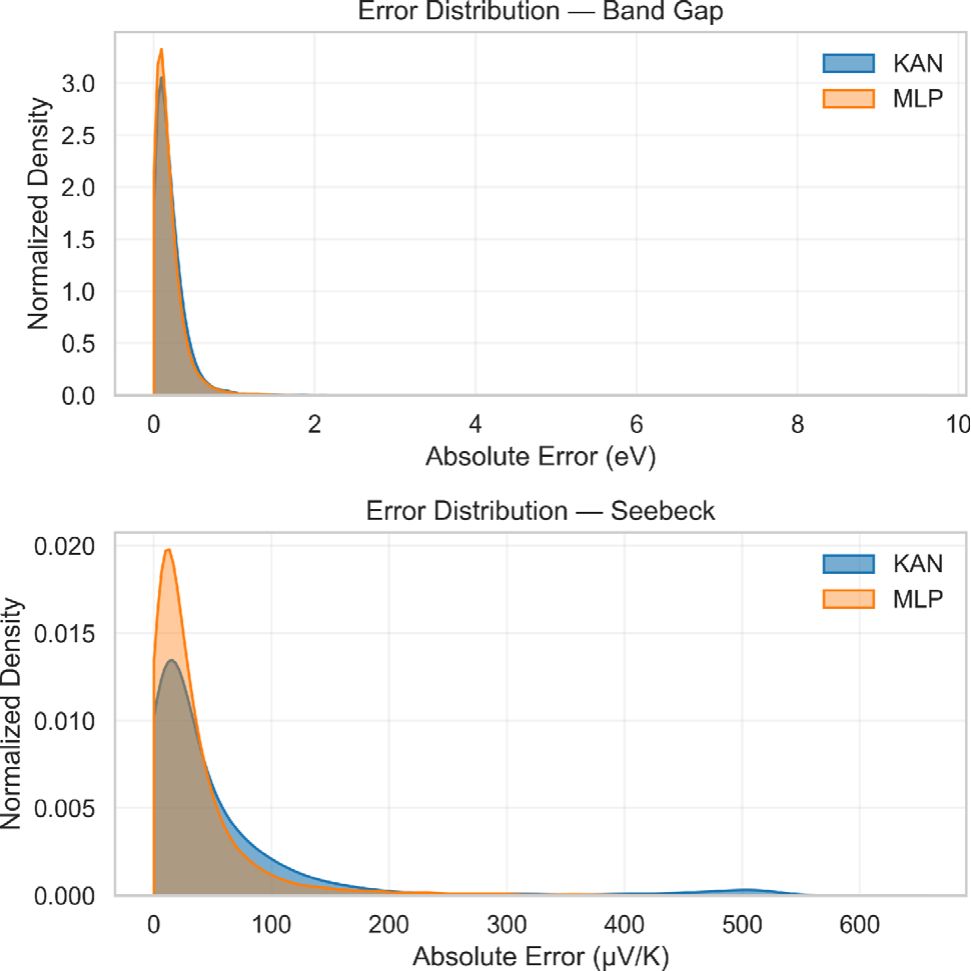
Kernel density estimates (KDE) of absolute prediction errors for (top) electronic band gap and (bottom) Seebeck coefficient, comparing Kolmogorov–Arnold Networks (KAN, blue) and Multilayer Perceptrons (MLP, orange).

The predictive behaviour of the KAN and MLP architectures was evaluated across the entire dataset using both global and element-resolved diagnostics. Figure 7 compares the absolute error distributions for electronic band gap and Seebeck coefficient. In both properties, the error density is sharply centred at low values, confirming that both models achieve consistent regression behaviour across most compounds. The KAN exhibits a slightly lower central peak and a small secondary peak at large error values compared with the MLP, indicating a broader but more stable distribution.

**Fig. 8:**
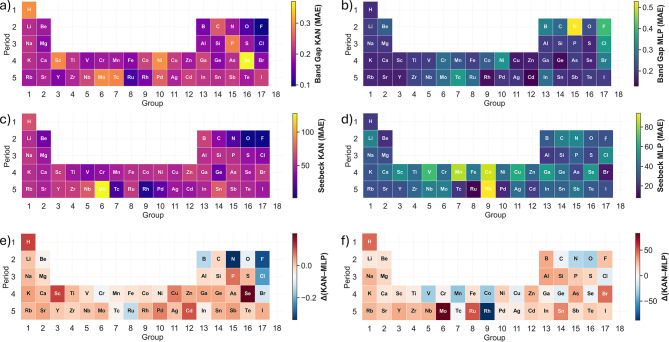
Element-resolved mean absolute error (MAE) maps for band gap and Seebeck predictions. Each periodic-table cell represents the mean error across all compounds containing that element. Panels (a-b) show element-wise MAE for the KAN and MLP band-gap predictions, while (c-d) report analogous values for Seebeck coefficients. Panels (e-f) display the inter-model differences $\Delta$(KAN-MLP), where blue tones indicate superior KAN performance. KAN achieves more uniform accuracy across s-, p-, and d-block elements, while MLP exhibits stronger element-dependent variability, particularly for transition metals and chalcogens.

To identify the chemical origins of uncertainty, the mean absolute error (MAE) and inter-model difference $\Delta = \textrm{Value}_\textrm{KAN} - \textrm{Value}_\textrm{MLP}$ were averaged for each element across all compounds containing that species (Fig. 8). Here, $\textrm{Value}_\textrm{KAN}$ and $\textrm{Value}_\textrm{MLP}$ denote the property values predicted by the Kolmogorov–Arnold Network and the Multilayer Perceptron, respectively, and $\Delta$ represents their pointwise difference. Both properties reveal systematic chemical trends.

For band gaps, the largest errors are observed in compounds containing Se (KAN) or N (MLP), as well as those including late transition metals (Fe, Co, Ni, Cu) and *p*-block elements. On average, the KAN performs best for halogens, confirming reliable learning in simple ionic regimes, whereas the MLP performs worst in these same groups. Conversely, in alkali and alkaline-earth metals, the MLP achieves slightly lower errors.

For Seebeck coefficients, the overall trend differs from that of the band gap: the largest errors occur for Mo (KAN) and Rh (MLP). In general, the MLP exhibits marginally lower element-wise variance. Consequently, the difference maps $\Delta (\text {KAN--MLP})$ are predominantly red (positive), indicating that the MLP generally produces lower element-averaged MAEs. However, both models maintain low absolute errors, and the element-wise differences are small relative to the overall prediction magnitude, confirming that generalisation is robust for both architectures across most elements.

**Table 5:** Tail error comparison on the held-out test set. Percentiles are computed from the absolute residual distribution.

Property	Model	95% error	99% error	Max error
Band gap (eV)	KAN	0.52	0.99	9.61
Band gap (eV)	MLP	0.54	1.02	9.43
Seebeck ($\mu$V/K)	KAN	122.0	289.9	657.5
Seebeck ($\mu$V/K)	MLP	186.3	505.5	611.2

To quantify robustness beyond mean accuracy metrics, we evaluated the upper percentiles of the absolute residual distributions on the held-out test set (Table 5). While both architectures exhibit comparable maximum errors, differences emerge in the high-percentile regime. For band-gap prediction, KAN yields marginally lower 95th and 99th percentile errors relative to MLP, indicating slightly improved stability in the upper tail of the error distribution. The effect is more pronounced for Seebeck prediction, where KAN reduces both the 95th and 99th percentile errors compared with MLP.

These global statistics are consistent with the structured disagreement analysis reported in Table 7, where KAN exhibits more coherent behaviour across electronically complex compounds. Taken together, the percentile-based tail analysis and the compound-level disagreement patterns indicate that, although mean absolute errors are similar between architectures (Tables 1-3), the spline-based functional representation of KAN promotes smoother and more stable behaviour in challenging regions of descriptor space.

Importantly, these differences arise without the imposition of explicit physics-based constraints or post hoc filtering. The observed moderation of extreme deviations therefore reflects the intrinsic inductive bias of the spline-based architecture rather than dataset curation or manual regularisation.

#### Origin of predictive uncertainty

By comparing the elements with the largest and smallest mean absolute errors ($\text {MAE}_{\text {avg}}$ across KAN and MLP) in band-gap prediction, we can trace the physical origin of these differences and clarify how KAN and MLP handle distinct bonding and electronic regimes.

*a. High-Uncertainty Elements (Large* $\text {MAE}_{\text {avg}}$ *):* The elements exhibiting the highest prediction errors (e.g., $\text {N, C, F, P, Se}$ in the *p*-block; $\text {Ni, Co, Mo, Tc}$ in the *d*-block) share two defining characteristics:

1. **Covalent directionality and localisation:** Light *p*-block elements ($\text {N, C, F}$) form highly directional $\sigma$ and $\pi$ bonds, requiring the model to resolve intricate, non-linear dependencies on bond angles and orbital overlap. This leads to strong localisation of valence states and sensitivity of the band gap ($E_g$) and Seebeck coefficient (*S*) to small structural deviations. In these regimes, KAN predictions exhibit more gradual spatial variation of element-resolved errors across adjacent groups in the periodic table (Fig. 8), consistent with a smoother functional response in descriptor space. This behaviour is further supported by the tail-percentile analysis (Table 5), where KAN displays reduced 95th and 99th percentile errors for band-gap prediction and a moderated upper tail for Seebeck coefficients. We note that this smoothing effect is most evident in chemically sparse regions of the training distribution, particularly for light *p*-block elements where directional bonding amplifies sensitivity to small descriptor perturbations. In densely sampled or chemically simple regimes, both KAN and MLP display comparable continuity.
2. **Partially Filled** *d***-Shells and Correlation:** Mid-to-late transition metals ($\text {Ni, Co, Mo, Tc}$) introduce partially filled *d*-orbitals, which leads to strong $\text {d--d}$ electron correlation effects (Mott-Hubbard physics) or highly complex $\text {p-d}$ hybridization near the Fermi level. These phenomena are intrinsically difficult for standard machine learning models, whose embeddings struggle to fully parameterize the non-local and dynamic electronic interactions. In the mid-to-late transition metal region (e.g., Fe, Co, Ni, Cu), which is characterised by partially filled *d*-shells and strong electronic correlation, the element-resolved error maps (Fig. 8) reveal more uniform inter-element variation for KAN compared with MLP. Approximate element-averaged values (see SI) indicate a smaller inter-element MAE spread for KAN in both band gap and Seebeck predictions. This behaviour is further supported by the PCA projection analysis (Fig. 10), where high-disagreement compounds cluster in regions associated with chemically complex transition-metal systems. The concentration of disagreement in these regions suggests that architectural differences primarily emerge in electronically correlated regimes rather than in simple ionic materials. This smoother chemical-space variation is consistent with the spline-based functional representation of KAN, which enforces continuity in the learned mapping. In contrast, MLP displays sharper localised increases in error for specific transition-metal elements, reflecting higher sensitivity to descriptor perturbations in electronically correlated regimes.

*b. Low-Uncertainty Elements (Low* $\text {MAE}_{\text {avg}}$*):* Conversely, the elements associated with the lowest errors (e.g., $\text {Mg, Sr, Rb}$ in the *s*-block; $\text {Zn, Cd}$ in the *d*-block; $\text {O, S, Ge}$ in the *p*-block) are characterized by electronic simplicity and predictability:

1. **Simple Ionicity:** Alkali and alkaline-earth metals drive simple ionic bonding in wide-gap insulators. Their contributions to the band structure are often far from the Fermi level, resulting in predictable band gaps dominated by well-behaved $\text {s}$ and $\text {p}$ states.
2. **Full or Simple Shells:** Elements like $\text {Zn}$ and $\text {Cd}$ possess a full $d^{10}$ shell, rendering their chemistry and electronics less complex than those of their neighbors with partially filled *d*-bands. Likewise, the common, highly represented structures formed by $\text {O}$ and $\text {S}$ (simple oxides and sulfides) provide abundant and consistent training examples, enabling robust model learning.

The element-resolved maps do not indicate uniformly lower mean errors for KAN across all species; rather, the distinguishing feature is reduced localised error spikes and more coherent error gradients across chemically related elements. This structured behaviour contrasts with the sharper element-dependent variability observed in the MLP maps, particularly within light *p*-block and correlated *d*-block systems.

These observations suggest that the spline-based parameterisation of KAN imposes continuity in the learned mapping, mitigating abrupt prediction shifts in sparsely sampled or electronically complex regions of chemical space^31,56,57^.

This analysis suggests that the primary challenge for the predictive models lies not in simple compositional variety, but in regions of the periodic table defined by high electronic correlation and highly directional, non-linear bonding characteristics. This robustness in modeling highly nonlinear functional dependencies makes KAN uniquely suited for the subsequent step of symbolic regression, where stable and smooth functional forms are crucial for physical interpretability.

**Fig. 9:**
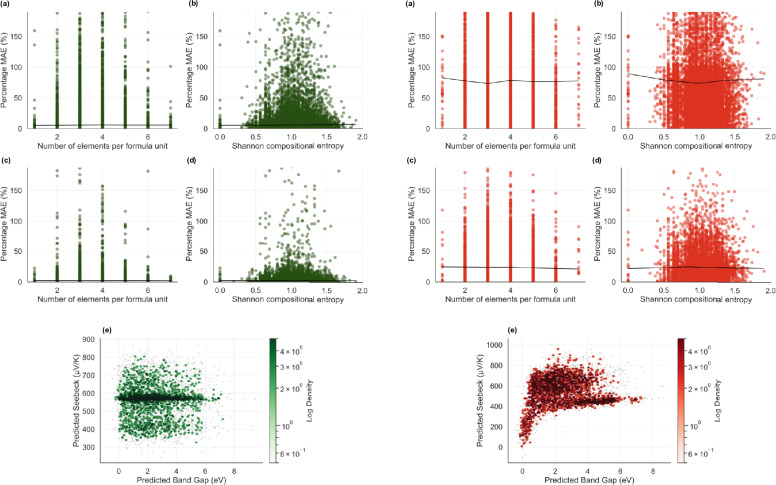
Error behaviour across compositional complexity and electronic regimes. Panels left (green) and right (red) compare the performance of KAN and MLP models, respectively, as functions of compositional entropy and elemental diversity, showing that both generalise well across simple and high-entropy materials. At high complexity, MLP predictions become more scattered, whereas KAN maintains smoother, composition-invariant accuracy. The lower panels illustrate the relationship between Seebeck coefficient and band gap: MLP follows a single dominant Pisarenko-like trend, while KAN displays a broader but more continuous mapping that captures multiple transport regimes within a unified functional representation.

To probe whether chemical complexity correlates with predictive uncertainty, we analysed the percentage MAE as a function of both the number of elements per formula unit and the Shannon compositional entropy, $S_\textrm{chem} = -\sum _i x_i \ln x_i$, where $x_i$ denotes the fractional atomic concentration (Fig. 9). Spearman’s rank correlation coefficient indicates no statistically significant monotonic dependence between model error and compositional entropy ($|\rho | \le 0.01$ for all targets), confirming the absence of a global increasing or decreasing trend across the entropy range. Nevertheless, the density maps reveal a weak non-monotonic structure. In particular, KAN errors exhibit a modest elevation around intermediate entropy values ($S_\textrm{chem} \approx 1$), while remaining comparatively lower at both low and high entropy extremes. This intermediate-entropy region corresponds to moderately complex compositions, where competing bonding motifs and electronic hybridisation effects coexist.

Importantly, this localised elevation does not constitute a systematic monotonic dependence on entropy. Error magnitudes remain bounded across the full compositional spectrum, and no progressive degradation of accuracy is observed with increasing chemical complexity. Both architectures therefore generalise robustly across simple, moderately complex, and high-entropy systems, with only mild local variations in uncertainty.

When the Seebeck coefficient is plotted against the band gap (Fig. 9, bottom panels), the MLP architecture exhibits a weak positive global correlation (Spearman $\rho = 0.11$), broadly consistent with the well-known tendency for larger band gaps to suppress bipolar conduction and enhance thermopower in semiconductors. This behaviour is qualitatively related to the classical Pisarenko relation, which describes the dependence of the Seebeck coefficient on carrier concentration under fixed band structure assumptions^58,59^.

In contrast, the KAN model exhibits negligible global monotonic correlation ($\rho = -0.02$), indicating that it does not enforce a simple one-parameter scaling between band gap and thermopower. Instead, the learned mapping distributes Seebeck values across multiple regimes at comparable band gaps, reflecting the heterogeneous chemical composition and varying transport mechanisms represented in the dataset.

The Pisarenko relation strictly applies under assumptions of parabolic bands and fixed scattering mechanisms, whereas the present dataset includes chemically diverse systems with varying carrier types, effective masses, and scattering regimes. The absence of a strong monotonic trend in the KAN predictions therefore does not imply a failure to capture physical behaviour; rather, it may reflects the breakdown of simple Pisarenko scaling across a heterogeneous materials space.

A more targeted comparison was performed by analysing the 10% of compounds with the largest KAN–MLP prediction differences.

Statistical analysis (Table 7) shows that high-disagreement band-gap compounds possess slightly larger average gaps but comparable compositional characteristics. This observation is consistent with the entropy-bin analysis (Fig. 9), which indicates no systematic monotonic trend between complexity and prediction error. Importantly, in the high disagreement region, KAN performs consistently better than MLP with respect to the true target values. To quantify the magnitude of these divergences, we computed the mean absolute errors within the top 10% KAN–MLP disagreement subset (Table 6). For band-gap prediction, the mean error of KAN across these compounds is 0.38 eV, whereas the corresponding MLP error reaches 1.12 eV, representing an $\sim$3-fold increase. For Seebeck prediction, the mean KAN error remains 48.3 $\mu$V/K, while MLP exhibits a substantially larger mean error of 187.6 $\mu$V/K, corresponding to a $\sim$4-fold difference.

These results indicate that, in the most challenging regimes of chemical and electronic complexity, the divergence between architectures is not marginal but amplified for the MLP in specific extreme cases. In contrast, the spline-based KAN representation maintains bounded, physically plausible predictions, avoiding multi-electronvolt band-gap deviations and sign-inverted thermopower values. This quantitative analysis confirms that the architectural inductive bias of KAN moderates extreme extrapolation behaviour rather than merely improving average performance metrics.

**Table 6:** Quantitative comparison within the top 10% KAN–MLP disagreement subset. Values represent mean absolute errors computed from the worst-disagreement compounds shown in Table 7.

Property	MAE$_\textrm{KAN}$	MAE$_\textrm{MLP}$	Ratio (MLP/KAN)
Band gap (eV)	0.38	1.12	$\sim$3
Seebeck ($\mu$V/K)	48.3	187.6	$\sim$4

The convergence of these analyses establishes a few key findings:

1. The largest inter-model divergences occur in wide-bandgap systems with strong orbital localisation and low carrier density near the band edges.
2. KAN exhibits more spatially coherent error distributions across chemically related elements (Fig. 8) and reduced high-percentile residuals in band-gap prediction (Table 5), indicating enhanced stability in sparsely sampled or highly nonlinear regimes. In contrast, MLP predictions show stronger element-specific variability and sharper local deviations.
3. KAN more effectively suppresses high-error outliers whereas MLP preserves global monotonic trend but may exhibit local sign errors.

Overall, the results demonstrate that KAN’s continuous functional representation yields greater robustness and compositional invariance across the descriptor manifold. Importantly, this robustness does not scale monotonically with compositional entropy, consistent with the entropy analysis discussed above. Instead, the observed stability emerges as a structural property of the spline-based mapping in regions characterised by correlated electronic features rather than by compositional complexity alone^60,61^.

**Fig. 10:**
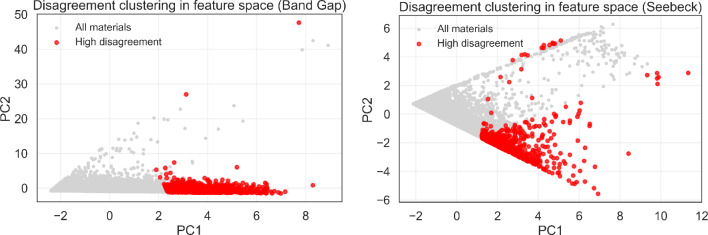
Principal component projections of the Crystalformer embeddings for (left) band-gap and (right) Seebeck datasets. Red points highlight the 0.2% tail of highest KAN–MLP disagreement.

To identify specific regions of feature space associated with large inter-model discrepancies, the Crystalformer embeddings were projected onto their first two principal components (PC1 and PC2; Fig. 10). This two-dimensional feature map shows the entire materials dataset as grey points and the compounds with the highest KAN–MLP disagreement as red points. Distinct clusters emerge in chemically complex regions of descriptor space, whereas the main data density remains concentrated near the origin ($\text {PC}1 \approx 0, \text {PC}2 \approx 0$), corresponding to the chemically simplest and most abundant training instances. The compounds where KAN and MLP exhibit the largest disagreement in band-gap prediction form a compact, displaced cluster ($\text {PC}1 \ge 2$), indicating that failure modes are not random but concentrated in specific regions of the learned descriptor manifold. As discussed in the compositional entropy analysis (Fig. 9), no statistically significant monotonic dependence between model error and chemical complexity is observed.

The disagreement clustering for Seebeck prediction shows a greater degree of dispersion than the band-gap failure modes (Fig. 10). While still displaced along $\text {PC}1$ ($\text {PC}1 \ge 1.5$), the Seebeck failure modes spread considerably across the $\text {PC}2$ axis, forming a bifurcated structure extending into both positive and negative $\text {PC}2$ space. This increased $\text {PC}2$ variance indicates that the mechanisms governing *S* prediction are more acutely sensitive to chemical/structural variations captured by $\text {PC}2$ than the mechanisms governing band-gap prediction. This property-specific dispersion suggest that thermoelectric response is governed by regime-dependent transport behaviors that become numerically separable in the high-complexity regions of chemical space.

#### Interpretability and physical consistency.

The improved stability of KAN across chemically and electronically complex regimes arises from its architectural inductive bias: a locally adaptive spline basis with explicit smoothness regularisation. Each edge function is parameterised by cubic B-splines ($k=3$) with $C^2$continuity, ensuring continuity of both function values and first derivatives across knot boundaries^60,61^. This contrasts with ReLU-based MLPs, where piecewise-linear activations permit abrupt slope changes and higher local curvature in sparsely sampled regions.

In addition, the entropy-based regularisation term introduced during training penalises excessive concentration of spline coefficients, effectively constraining high-frequency oscillations in the learned functional mapping. This regularisation acts in function space by constraining spline curvature and coefficient concentration, thereby limiting high-frequency oscillations in sparsely sampled regions of descriptor space.

This theoretical expectation is empirically consistent with the residual and element-resolved analyses discussed above. For band-gap prediction, KAN exhibits slightly lower 99th-percentile error (0.99 eV) compared to MLP (1.02 eV), indicating moderated tail behaviour. A similar trend is observed for Seebeck prediction, where KAN shows reduced extreme residuals relative to MLP. Element-resolved MAE maps (Fig. 8) further reveal smoother inter-element gradients for KAN across chemically related transition-metal series. In contrast, MLP displays more localised error spikes, indicative of higher local variance in descriptor space. Together, these observations support the interpretation that spline-based functional regularisation promotes controlled interpolation across chemically correlated regions.

These observations align with theoretical and approximation-theoretic analyses of spline-based function representations.

In the context of Kolmogorov–Arnold Networks, Liu *et al.*demonstrated that the edge-wise spline parameterisation induces an adaptive functional regularisation that improves stability under sparse sampling and reduces overfitting relative to piecewise-linear activations^31^. Recent theoretical analysis further shows that spline-based KAN architectures exhibit improved approximation smoothness and bounded variation in low-density regions of input space compared with standard ReLU networks^57^.

Empirically, this theoretical behaviour is reflected in the reduced high-percentile residuals reported in Table 5, where KAN achieves lower 99th-percentile errors in band-gap prediction and moderated tail behaviour in Seebeck prediction. The agreement between approximation theory, recent KAN analyses, and the observed error-tail statistics provides quantitative support for the interpretation of KAN as enforcing functional regularisation in descriptor space.

On the held-out test set, KAN produces a limited number of Seebeck sign inversions, whereas the MLP exhibits a number cases in which the predicted carrier type is reversed (see SI). Similarly, the MLP generates a larger number of physically implausible band-gap predictions (negative values), while KAN reduces such violations. These results suggest that the spline-based functional continuity of KAN suppresses abrupt regime switching and mitigates extrapolative artefacts observed in the MLP model.

These compounds serve as diagnostic markers: they pinpoint precisely where KAN’s locally adaptive, continuous representation enforces physical plausibility while MLP’s discrete function approximation breaks down. All materials shown are from the upper 0.2% tail of the error distribution, representing the most challenging test cases for generalization.

Across all the worst-performing compounds, the KAN consistently delivers smaller absolute errors than the MLP, confirming that its spline-based formulation not only improves average stability but also dominates in the most challenging prediction regimes.

A concise summary of these critical cases is provided in Table 7.

**Table 7:** Representative materials exhibiting the largest KAN–MLP prediction discrepancies. Each compound is listed with the model providing the most accurate result and a brief description of its key physical or chemical characteristics. These cases illustrate the diversity of bonding types and electronic regimes represented in the dataset.

*Band-gap predictions*		
**Compound**	**Best model**	**Main material characteristics**
TmMg$_5$	KAN	Intermetallic compound with partially filled *f*-shell (Tm) and metallic bonding; narrow-gap or semimetallic behaviour.
CaIn$_2$	KAN	Polar semimetal with covalent In–In chains and metallic Ca layers; features delocalised *p*-states.
Ga$_3$Co	KAN	Metallic compound with strong *p*–*d* hybridisation between Ga and Co; representative of covalent–metallic alloys.
SrCaI$_4$	KAN	Highly ionic mixed halide containing alkaline-earth metals; wide-gap insulator dominated by ionic bonding.
Er$_2$Si$_3$Rh	KAN	Complex intermetallic silicide with mixed *d*–*f* electron character and heavy-atom correlation.
B$_2$Mo	KAN	Covalent boride with strong B–B $\pi$ bonding and Mo *d*-state hybridisation; narrow-gap semimetal.
SbH(OF$_3$)$_2$	KAN	Molecular-like oxyfluoride with highly electronegative ligands; polar covalent bonding and strong anion asymmetry.
BaHfMo	KAN	Double perovskite-type oxide with mixed *d*-electron occupation (Hf/Mo); moderate band gap and ionic–covalent bonding.
Li$_4$Fe$_3$Co$_3$(WO$_8$)$_2$	KAN	Multimetallic oxide with spin-polarised Fe/Co *d*-states and strong electron correlation typical of transition-metal oxides.
*Seebeck predictions*		
**Compound**	**Best model**	**Main material characteristics**
CSe$_2$S$_2$N$_2$(OF)$_3$	KAN	strong covalency and high electronegativity contrast; prone to large carrier localisation.
AuSe	KAN	Narrow-gap semimetal with strong spin–orbit coupling and hybridised *d*–*p* orbitals.
Na$_5$Fe$_2$P$_2$(CO$_7$)$_2$	KAN	Polyanionic phosphate–carbonate complex containing Fe *d*-states; polaronic conduction and mid-gap thermopower.
Sm$_3$S$_4$	KAN	Narrow-gap rare-earth sulfide with strongly correlated *f*-electrons; known for high thermoelectric response.
HgTe	KAN	Small-gap semimetal and topological material; strong *p*–*d* mixing and inverted band structure.
SmAs	KAN	Rare-earth pnictide with mixed covalent–metallic bonding; *f*–*p* hybridisation near the Fermi level.
Te$_4$MoBr	KAN	Layered telluride–bromide system with mixed ionic and covalent bonding; heavy-atom conduction bands.
Ti$_3$Cu$_2$Te(PO$_4$)$_6$	KAN	Multimetallic chalcogenophosphate with correlated *d*-electron transport and narrow conduction band.
Ba$_5$Re$_3$ClO$_{15}$	KAN	Rhenium-based perovskite oxide with extended *d*-band manifold and polar halide coordination.

#### KAN attribution scores

To assess the relevance of individual descriptors we used the built-in attribution analysis available in the Kolmogorov–Arnold Network framework. In KANs, each edge between nodes carries an adaptive spline function that directly maps input values to activations. During attribution, the network is first evaluated on a representative dataset to record the activations of all spline functions. The contribution of each input descriptor to the final output is then quantified by aggregating the absolute magnitudes of the learned spline functions along all paths that connect the input to the output node. Formally, the attribution score for descriptor $x_i$ is defined as

9 $$\begin{aligned} S_i = \frac{1}{Z}\,\sum _{p \in \mathcal {P}(i \rightarrow y)} \prod _{(u \rightarrow v) \in p} \big | f_{uv}(a_u) \big |, \end{aligned}$$

where $\mathcal {P}(i \rightarrow y)$ denotes the set of all directed paths from input $x_i$ to the output node *y*, $f_{uv}$ is the spline function along edge $(u \rightarrow v)$, $a_u$ is the activation of node *u*, and *Z* is a normalisation factor ensuring $\sum _i S_i = 1$. This procedure yields a feature attribution score for every descriptor, which can be interpreted as a normalised measure of its overall influence on the target prediction. Unlike gradient-based sensitivities, which reflect local responsiveness of the output to infinitesimal perturbations, KAN attribution scores incorporate the full functional form of the spline edges, and thus provide a more global estimate of descriptor importance consistent with the symbolic structure of the trained model.

### Symbolic representation

The first step in achieving symbolic interpretability is the identification of the most influential input descriptors. This was carried out using the KAN attribution score (see Fig. 12 and Fig. 14), which directly quantifies the sensitivity of the predicted property to each input. The descriptors with the highest attribution scores were selected for further analysis: $(x_{39},x_{68})$ for band gap and $(x_{39},x_{83})$ for the Seebeck coefficient. To assess whether the dominant descriptors encode direct physical proxies (e.g., carrier concentration), we computed Pearson and Spearman correlations between all 128 CrystalFormer features and each target property. The strongest observed linear correlations remain modest ($|r| < 0.20$ across all features; see SI). Descriptor $x_{39}$ ranks among the top correlated features, but its association with band gap and Seebeck remains weak-to-moderate (e.g., $r \approx 0.15$–0.17).

These values indicate that $x_{39}$ cannot be interpreted as a direct linear proxy for carrier concentration. Rather, its relevance emerges through nonlinear multivariate interactions captured by the spline-based functional representation of the KAN architecture.

**Fig. 11:**
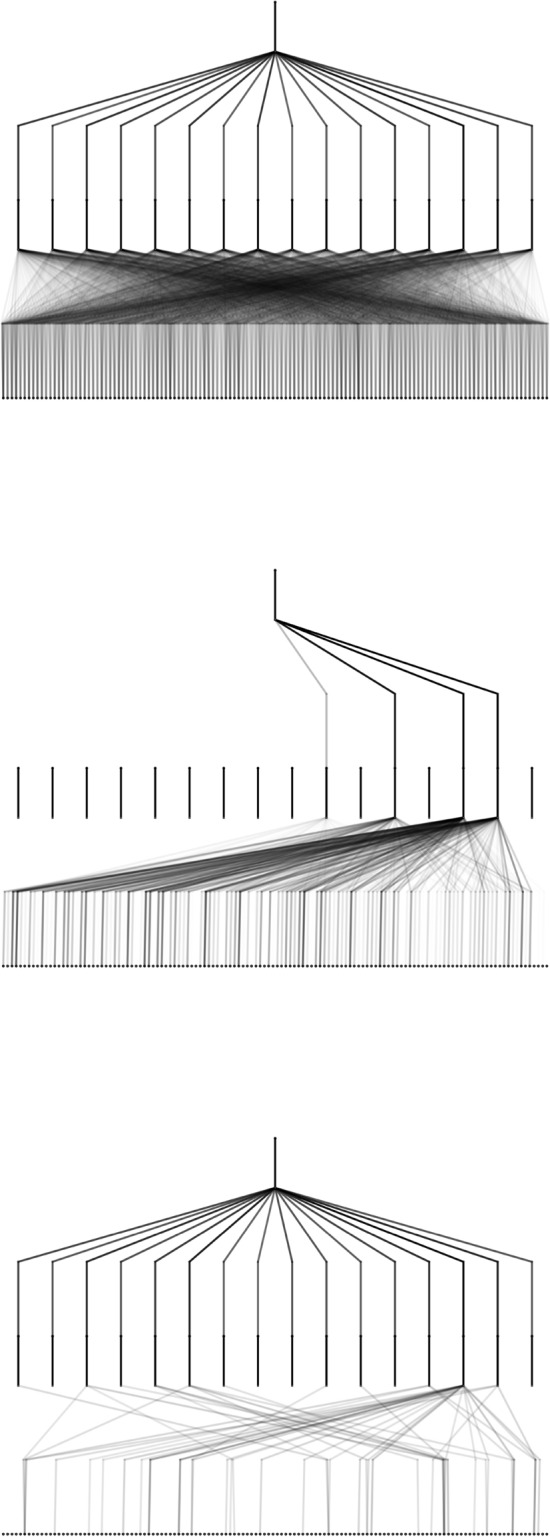
Kolmogorov–Arnold Network architectures. Top: pre-training fully connected architecture. Middle: Band Gap; Bottom: Seebeck coefficient. The architectures were obtained after training to the performance levels reported in Tables 3. The visualisations highlight how only a subset of edges contributes significantly to the learned structure–property mapping. This sparsity enables pruning of redundant connections, simplifying the network while retaining predictive accuracy and interpretability.

Redundant edges and nodes with negligible contribution were pruned. For each input feature, the input-hidden edge relevance was computed as the product of the spline coefficients’ magnitude with associated scaling parameters. Node relevance was then defined as the product of the total incoming edge strength and the strength of the outgoing connection to the output. This definition ensures that a node is considered important only if it integrates significant contributions and transmits them effectively. Edges with attribution scores below 0.02 were discarded. After pruning, the simplified models retained predictive performance within $R^2$ variations of 0.02, while revealing sparse, interpretable subnetworks (see Fig. 11). The index set $\mathcal {J}$ identifies the subset of active hidden nodes retained after pruning, each contributing a simplified symbolic component $\phi _j$ to the reconstructed analytic expression.

To visualise the learned structure–property mapping, we restricted attention to the two most relevant descriptors for each target. The general pre-activation of a hidden node *j* can be expressed as

10 $$\begin{aligned} s_j(x_a,x_b) \;=\; \sum _{m=1}^{16} w^{(a)}_m\, g^{(a)}_m(x_a) \;+\; \sum _{n=1}^{16} w^{(b)}_n\, g^{(b)}_n(x_b), \end{aligned}$$

where $x_a,x_b$ are the two selected descriptors, $g^{(a)}_m,g^{(b)}_n$ are the symbolic edge functions, and $w^{(a)}_m,w^{(b)}_n$ the corresponding weights (either uniform or proportional to $R^2$).

The hidden activation is then

11 $$\begin{aligned} h_j(x_a,x_b) = \phi _j\!\big (s_j(x_a,x_b)\big ), \end{aligned}$$

where $\phi _j$ is the symbolic hidden–output activation. Summing over all active hidden units $\mathcal {J}$ gives the two-descriptor surrogate for the target property:

12 $$\begin{aligned} y(x_a,x_b) \;=\; \sum _{j\in \mathcal {J}} h_j(x_a,x_b). \end{aligned}$$

**Fig. 12:**
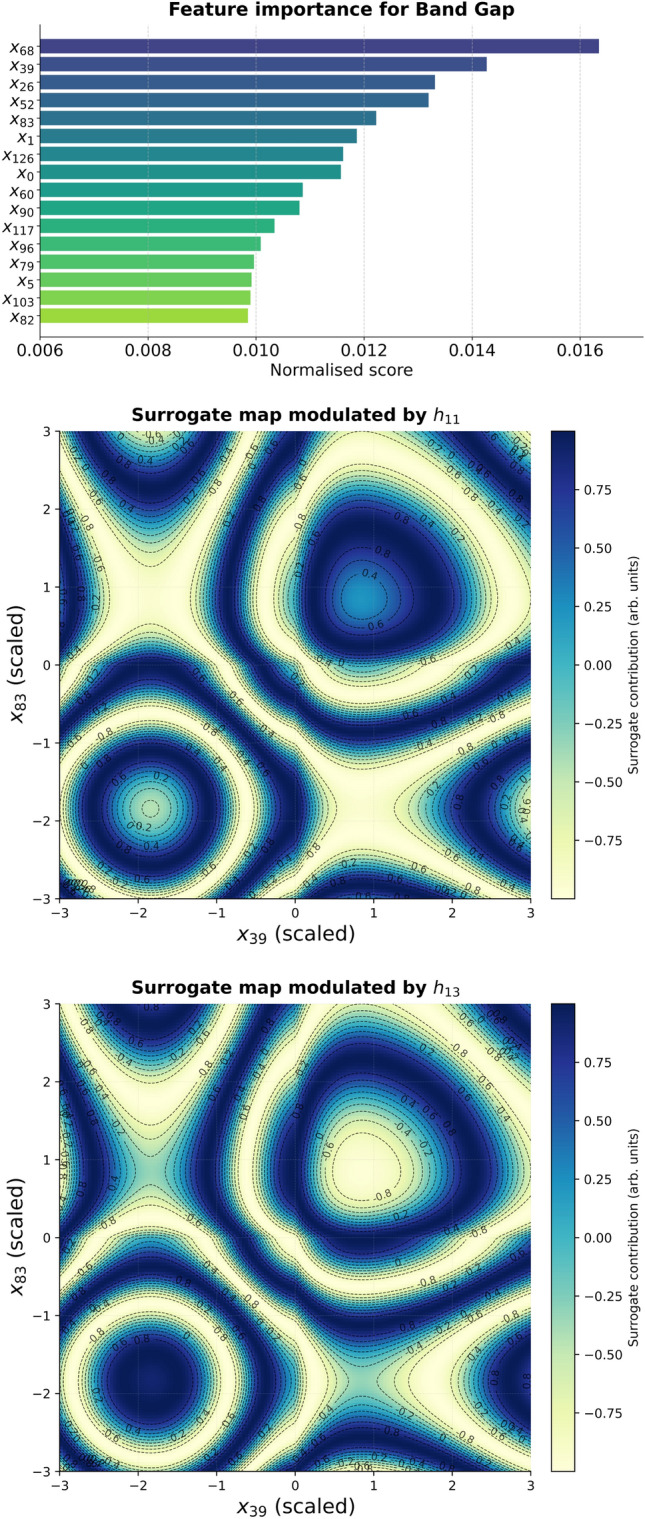
Top: band gap feature attribution scores computed using the KAN model. The ten descriptors with the highest attribution scores were selected for interpretability analysis. Middle and bottom: Fully modulated surrogate functions for the band gap model, constructed using the most relevant input descriptors.

This representation can be visualised as two-dimensional heatmaps, showing how the output varies with respect to pairs of descriptors while all other inputs are fixed at representative values (see Figs. 12 and 14).

A further simplification can be achieved by retaining only the most relevant hidden nodes for each descriptor pair. For the band gap model, analysis revealed that $x_{39}$ and $x_{68}$ dominate through nodes $h_9$ and $h_{13}$. For the Seebeck coefficient, $x_{39}$ and $x_{83}$ are channelled primarily through $h_{11}$ and $h_{13}$. Restricting the surrogate to these nodes yields compact expressions that preserve the dominant nonlinear mechanisms.

**Band gap surrogate:**

13 $$\begin{aligned} h_{9}(x_{39},x_{68})&= \cos \!\big (s(x_{39},x_{68})\big ), \end{aligned}$$

14 $$\begin{aligned} h_{13}(x_{39},x_{68})&= \sin \!\big (s(x_{39},x_{68})\,\big ), \end{aligned}$$

15 $$\begin{aligned} y_{\textrm{BG}}(x_{39},x_{68})&= h_{9}(x_{39},x_{68}) + h_{13}(x_{39},x_{68}). \end{aligned}$$

**Seebeck surrogate:**

16 $$\begin{aligned} h_{11}(x_{39},x_{83})&= \tanh \!\big (S(x_{39},x_{83})\big ), \end{aligned}$$

17 $$\begin{aligned} h_{13}(x_{39},x_{83})&= \big |\,S(x_{39},x_{83})\,\big |, \end{aligned}$$

18 $$\begin{aligned} y_{\textrm{S}}(x_{39},x_{83})&= h_{11}(x_{39},x_{83}) + h_{13}(x_{39},x_{83}). \end{aligned}$$

**Fig. 13:**
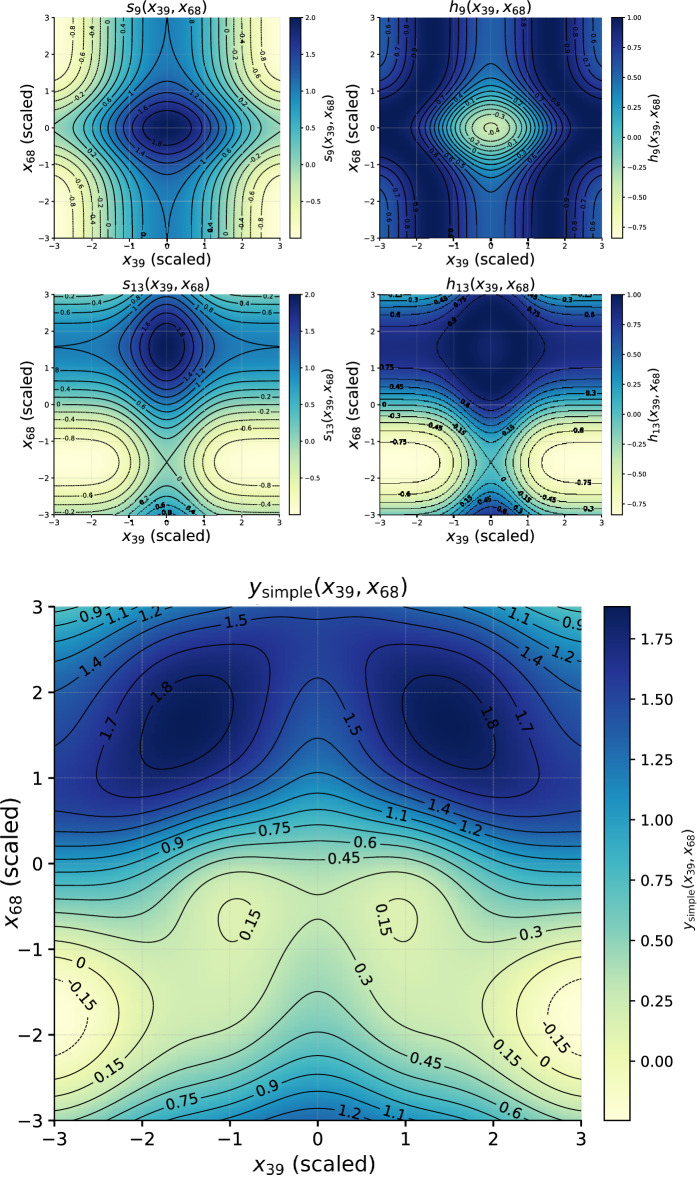
Simplified surrogate maps from the most relevant hidden nodes. Top: pre-activation ($s_{11}$, $s_{13}$) and post-activation ($h_{9}$, $h_{13}$). Bottom: combined surrogate output $y_{\textrm{BG}}(x_{39},x_{68})$.

These simplified surrogates were used to generate four heatmaps (two per property, one per node as shown in Figs. 13 and 15) together with the combined output maps. The node-specific maps highlight distinct nonlinear responses, while the combined maps represent the net predicted property. Together, they provide interpretable insight into how descriptor pairs control band gap and Seebeck coefficient. For completeness, Table 8 lists the symbolic fits obtained for edges connecting descriptor $x_{68}$ to the first hidden layer in the band gap model.

The heatmaps reveal cooperative effects between descriptors: for the band gap, oscillatory modulations from $x_{39}$ interact with Gaussian-like localisations from $x_{68}$, while for the Seebeck coefficient, trigonometric oscillations along $x_{39}$ combine with saturating and Gaussian, together with the sinusoidal oscillations responses of $x_{83}$. Such patterns qualitatively reflect the multivariate dependence of thermoelectric response on electronic structure descriptors. However, correlation analysis across all CrystalFormer features indicates that no single descriptor exhibits strong linear correlation with either band gap or Seebeck coefficient (maximum $|r| < 0.2$). This suggests that the learned behaviour does not arise from a direct one-to-one mapping between a specific descriptor and carrier concentration, but rather from nonlinear interactions among multiple correlated structural features captured by the spline-based KAN representation. While classical semiconductor models relate Seebeck enhancement to reduced carrier concentration and suppressed bipolar conduction, the present data-driven analysis shows that no single learned descriptor exhibits strong direct correlation with either band gap or Seebeck coefficient. The extracted symbolic relations therefore represent distributed, nonlinear descriptor interactions rather than explicit carrier-density mappings. The detailed symbolic approximations for the edges involving descriptors $x_{39}$ and $x_{83}$ are summarised in Table 9, providing explicit forms for the Seebeck coefficient surrogate.

These results demonstrate the strength of KANs for reverse engineering in materials design: they expose explicit functional forms that can guide electronic and thermoelectric optimisation. Nevertheless, the approach has limitations: two-dimensional projections cannot capture the full high-dimensional descriptor space, and hidden-node surrogates may not correspond to unique physical mechanisms. Future work should combine descriptor dimensionality reduction with KAN symbolic extraction, to obtain minimal, physically meaningful descriptor sets. This would allow KANs to realise their full potential as interpretable surrogates for structure–property mapping in materials science.

**Table 8:** Symbolic expressions fitted to the functions associated with the edges connecting input feature $x_{68}$ to the first hidden layer in the band gap prediction model. The coefficient of determination ($R^2$) quantifies the quality of each symbolic fit, and *c* denotes the corresponding function complexity.

Layer	In_idx	Out_idx	Function	$R^2$	*c*
0	68	0	gaussian	0.9165	3
0	68	1	abs	0.9297	3
0	68	2	abs	0.9535	3
0	68	3	sin	0.9923	2
0	68	4	gaussian	0.9876	3
0	68	5	sin	0.9894	2
0	68	6	cos	0.9750	2
0	68	7	sin	0.9548	2
0	68	8	cos	0.9648	2
0	68	9	sin	0.9933	2
0	68	10	gaussian	0.9911	3
0	68	11	gaussian	0.9881	3
0	68	12	cos	0.8568	2
0	68	13	sin	0.9856	2
0	68	14	cos	0.9637	2
0	68	15	abs	0.9951	3
0	39	0	sin	0.9907	2
0	39	1	sin	0.8074	2
0	39	2	cos	0.9824	2
0	39	3	sin	0.9841	2
0	39	4	cos	0.8525	2
0	39	5	abs	0.9931	3
0	39	6	gaussian	0.8167	3
0	39	7	abs	0.9664	3
0	39	8	cos	0.9866	2
0	39	9	gaussian	0.9285	3
0	39	10	sin	0.9917	2
0	39	11	gaussian	0.9848	3
0	39	12	abs	0.9938	3
0	39	13	gaussian	0.9896	3
0	39	14	cos	0.9428	2
0	39	15	sin	0.9375	2
1	0	0	abs	0.9866	3
1	1	0	cos	0.9696	2
1	2	0	abs	0.9087	3
1	3	0	tanh	0.9957	3
1	4	0	gaussian	0.9714	3
1	5	0	tanh	0.9833	3
1	6	0	tanh	0.8280	3
1	7	0	sin	0.9655	2
1	8	0	tanh	0.9961	3
1	9	0	cos	0.5921	2
1	10	0	gaussian	0.9877	3
1	11	0	gaussian	0.9984	3
1	12	0	cos	0.9949	2
1	13	0	sin	0.9992	2
1	14	0	sin	0.9801	2
1	15	0	abs	0.9801	3

**Fig. 14:**
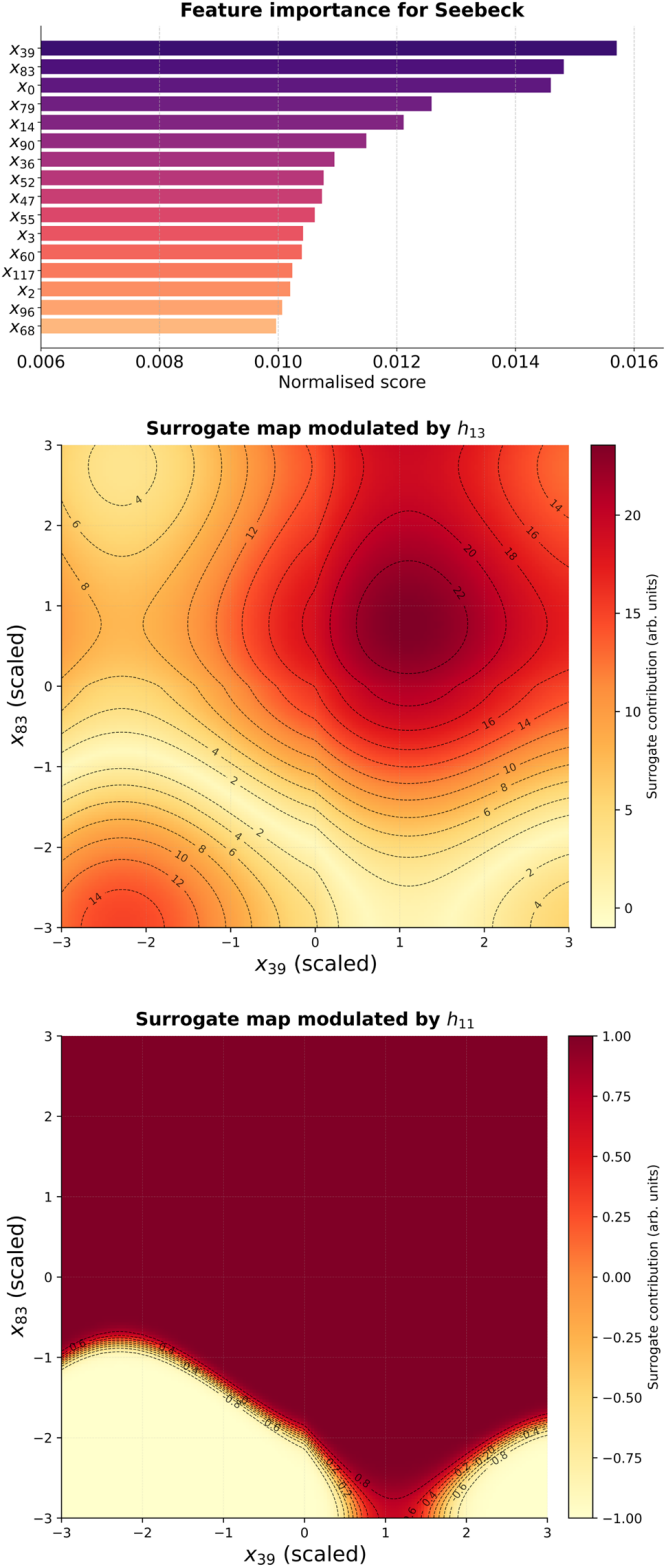
Top: Seebeck feature attribution scores computed using the KAN model. The ten descriptors with the highest attribution scores were selected for interpretability analysis. Middle and bottom: Fully modulated surrogate functions for the Seebeck coefficient model, constructed using the most relevant input descriptors.

**Table 9:** Symbolic expressions approximating the functions along edges connecting input features $x_{39}$ and $x_{83}$ to the first hidden layer in the Seebeck coefficient prediction model. The coefficient of determination ($R^2$) measures the fit accuracy, while *c* indicates the symbolic function complexity.

Layer	In_idx	Out_idx	Function	$R^2$	*c*
0	39	0	cos	0.9860	2
0	39	1	sin	0.8883	2
0	39	2	cos	0.9904	2
0	39	3	abs	0.9519	3
0	39	4	abs	0.9419	3
0	39	5	*x*	0.9464	1
0	39	6	sin	0.9880	2
0	39	7	cos	0.9582	2
0	39	8	cos	0.7517	2
0	39	9	abs	0.9604	3
0	39	10	sin	0.9631	2
0	39	11	sin	0.9922	2
0	39	12	cos	0.9742	2
0	39	13	sin	0.9916	2
0	39	14	cos	0.8838	2
0	39	15	cos	0.9856	2
0	83	0	*x*	0.8660	1
0	83	1	*x*	0.8896	1
0	83	2	cos	0.9444	2
0	83	3	sin	0.9659	2
0	83	4	sin	0.9918	2
0	83	5	cos	0.9922	2
0	83	6	cos	0.9658	2
0	83	7	cos	0.9842	2
0	83	8	cos	0.8703	2
0	83	9	cos	0.9800	2
0	83	10	abs	0.7747	3
0	83	11	cos	0.9934	2
0	83	12	*x*	0.9407	1
0	83	13	gaussian	0.9729	3
0	83	14	abs	0.8528	3
0	83	15	cos	0.9660	2
1	0	0	tanh	0.8438	3
1	1	0	tanh	0.7225	3
1	2	0	tanh	0.9376	3
1	3	0	cos	0.6911	2
1	4	0	arctan	0.9858	4
1	5	0	tanh	0.9463	3
1	6	0	tanh	0.8150	3
1	7	0	cos	0.5992	2
1	8	0	gaussian	0.8841	3
1	9	0	cos	0.9630	2
1	10	0	tanh	0.7758	3
1	11	0	tanh	0.9521	3
1	12	0	tanh	0.9884	3
1	13	0	abs	0.9843	3
1	14	0	tanh	0.9791	3
1	15	0	tanh	0.9377	3

**Fig. 15:**
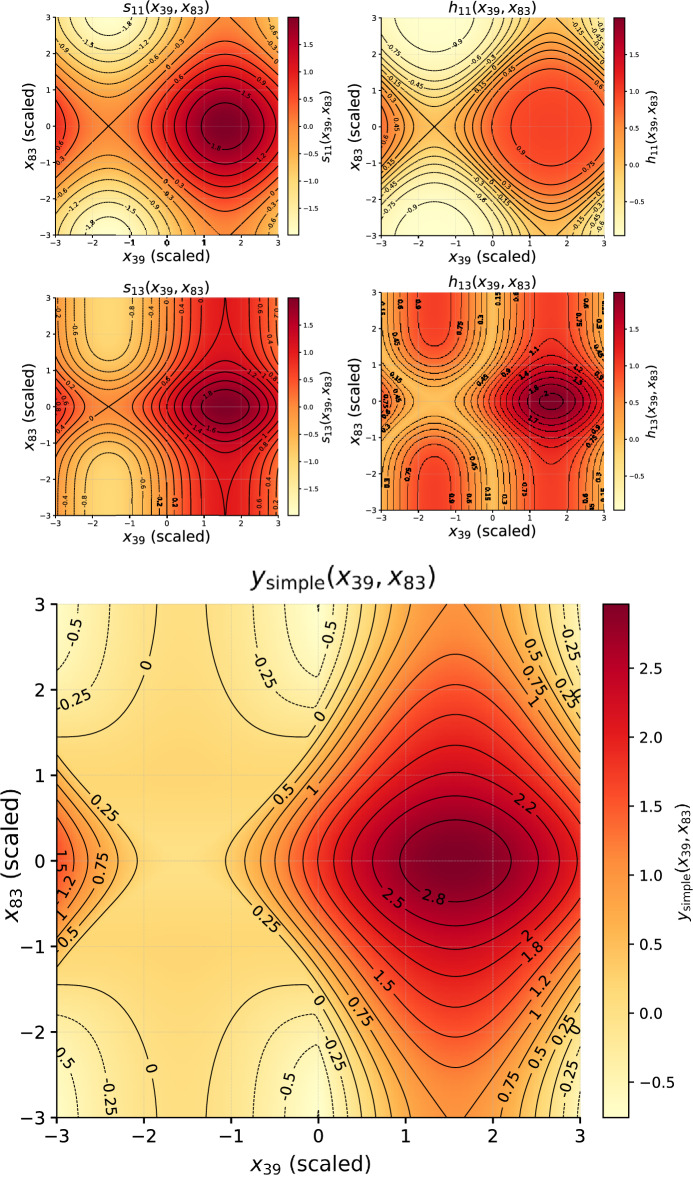
Simplified surrogate maps for Seebeck coefficient. Top: pre-activations ($s_{11}$, $s_{13}$) and activations ($h_{11}$, $h_{13}$). Bottom: combined surrogate output $y_{\textrm{S}}(x_{39},x_{83})$.

These surrogate maps capture how pairs of descriptors cooperate to shape the prediction, revealing nonlinear interactions that are difficult to infer from attribution scores alone. While limited to two-dimensional projections, they provide interpretable insights into the structure–property relationships encoded by the KAN. Future extensions combining descriptor-reduction methods with symbolic KAN analysis may enable extraction of compact, physically meaningful descriptor sets.

## Discussion

The results presented here address a longstanding tension in computational materials science between two competing paradigms for model discovery. Machine learning methods deliver impressive predictive accuracy but typically operate as black boxes, offering limited mechanistic insight into the underlying structure–property relationships. Sparse-optimisation and nonlinear-dynamics approaches, by contrast, yield explicit and interpretable mathematical equations, yet remain applicable only when the system admits an intrinsically sparse representation. Each paradigm thus carries fundamental limitations that constrain its utility for rational materials design. The KAN framework introduced here bridges these two paradigms by combining competitive predictive performance with functional transparency. Before discussing the broader implications of this capability, it is important to clarify the sense in which the term *inverse design*is used throughout this work. Here it refers to property-driven screening in the standard machine-learning sense, rather than direct atomic-structure synthesis. Specifically, the interpretable KAN models enable optimisation and sensitivity analysis within the learned descriptor space, which can then be coupled to existing materials databases or generative crystal models to propose candidate structures for subsequent first-principles validation. This two-step strategy—optimisation within a continuous representation followed by structure proposal and validation—is consistent with established inverse-design workflows in materials science^62–65^.

A key scientific contribution of our analysis lies in demonstrating that KANs can recover interpretable nonlinear functional structure in regimes where sparse linear or low-dimensional models are insufficient.

As shown in the correlation analysis (see SI), no individual feature exhibits strong linear dependence on the targets ($|r|<0.2$). We note that we do not claim intrinsic feature sparsity in the dataset; rather, the absence of strong pairwise correlations indicates that predictive structure arises from distributed nonlinear interactions across multiple descriptors. This weak single-feature dependence suggests that sparse linear selection strategies (e.g., LASSO-type regression or symbolic sparsity-based approaches) are not expected to identify a small set of strongly dominant linear descriptors with high explanatory power. In contrast, the spline-based KAN architecture captures distributed nonlinear interactions across multiple features while maintaining functional smoothness, enabling recovery of structured relationships without relying on explicit sparsity constraints. In functional space, there may exist infinitely many “shadowing” functions that reproduce the same dynamics without necessarily sharing the exact analytical form of the true governing equations. KANs naturally identify such shadowing functions, depending on architecture and regularisation, and thereby provide flexible but interpretable representations of the system’s behaviour. This suggests that KANs are not limited to symbolic regression in the narrow sense, but instead can map high-dimensional nonlinear processes into compact analytical surrogates that preserve dynamical fidelity. Concretely, the trigonometric and saturating symbolic forms extracted in §3.5 should be interpreted in this light: they represent one functionally consistent decomposition of the structure–property mapping rather than a unique or physically prescribed solution, and their value lies in the structured analytical access they provide to the descriptor–property surface.

Beyond their immediate predictive role, the scientific value of KANs lies in their ability to produce structured analytical mappings between descriptor space and target properties. In the present work, the symbolic dictionary was intentionally chosen to remain general and not to impose explicit physics-based functional forms.

The two-descriptor visualisations presented in Figs. 12,13,14 and 15 are simplified projections intended to illustrate dominant dependencies. However, the full multivariate analytical expression extracted from the trained KAN provides explicit functional control over the complete descriptor-property mapping.

We emphasise that complete reverse engineering from optimised descriptor values to atomic structures requires that the descriptor space be fully translatable into physically interpretable structural variables. Establishing such translation is beyond the scope of the present work. The aim of this study is to demonstrate the analytical power of KAN-derived maps, while future efforts will be required to couple these representations to explicitly physical descriptors for full inverse material design. Recent work has explored interpretable machine learning workflows for thermoelectric materials using feature-importance and SHAP-based analysis^66^. In such approaches, interpretability arises from post hoc attribution of compositional or structural descriptors within tree-based models. While highly valuable for trend identification, these methods do not typically yield explicit analytical surrogate functions that can be continuously inverted or manipulated. In contrast, the present KAN framework produces structured symbolic functional representations that approximate the full multivariate mapping between descriptor space and target properties. This distinction is important for reverse design: rather than identifying important features and manually exploring compositional changes, the KAN surrogate enables direct analytical exploration of the response surface in descriptor space. We therefore position the present work not as replacing feature-attribution workflows, but as extending interpretable modelling toward explicit functional reverse engineering.

The elementary function set therefore serves as a flexible approximation basis rather than an a priori physics-constrained model. By constraining or interpreting the learned symbolic functions in light of physical principles, one obtains structured and physically consistent representations that support mechanistic interpretation and hypothesis generation. While such representations do not by themselves establish causal mechanisms, they provide analytically tractable structure-property mappings that can guide controlled validation studies and physics-based model refinement. Overall, KANs therefore provide a principled bridge between classical approximation theory and modern machine learning.

*a. Accuracy of reference DFT data.*

The present work relies on the BoltzTraP transport dataset of Ricci *et al.*, which is constructed from high-throughput DFT calculations performed within semi-local exchange-correlation functionals and evaluated using the BoltzTraP code^36,67^.

It is well established that semi-local DFT functionals (e.g., PBE) systematically underestimate experimental band gaps, typically by 0.5–1.5.5.5 eV depending on the material class^68,69^. Therefore, the band-gap values used here should be interpreted as internally consistent DFT-level quantities rather than direct experimental observables.

The Seebeck coefficients in the Ricci dataset are computed within the constant relaxation-time approximation (CRTA), which assumes a uniform and energy-independent scattering time. While this approach captures qualitative transport trends, quantitative deviations from experiment can arise due to neglected scattering mechanisms, carrier lifetime variations, and temperature-dependent effects^70,71^. Reported discrepancies between BoltzTraP predictions and experimental Seebeck coefficients commonly range from 10–30%, depending on doping level and material complexity.

Importantly, the predictive errors of both KAN and MLP models are significantly smaller than the intrinsic uncertainty between DFT/CRTA predictions and experimental measurements. Consequently, the inverse-design analysis presented here operates within the DFT-BoltzTraP reference manifold. Any experimentally deployable optimisation would require subsequent validation using higher-level electronic-structure methods or direct experimental characterisation.

## Conclusion

We have demonstrated that Kolmogorov–Arnold Networks (KANs) offer a powerful and interpretable alternative to traditional machine learning approaches for predicting key thermoelectric properties, such as the Seebeck coefficient and electronic band gap. By leveraging their functional decomposition architecture, KANs deliver predictive performance comparable to standard multilayer perceptrons while providing symbolic surrogates that reveal the underlying structure-property relationships encoded in the data.

Our comparative analysis shows that KANs maintain high accuracy across both electronic and transport properties, despite their increased computational demands. The symbolic extraction pipeline enabled us to identify the most influential descriptors, prune redundant connections, and reconstruct compact analytical expressions that qualitatively align with known physical mechanisms. In particular, we highlighted how specific combinations of input features shape model outputs through interpretable hidden activations, offering insights that are otherwise inaccessible in conventional black-box networks.

These results position KANs as a practical framework for scientific model discovery and reverse engineering in materials science. By producing explicit functional maps between structural descriptors and target properties, KANs can guide the rational design of materials with tailored thermoelectric performance. Future extensions may integrate KANs with generative models, descriptor selection schemes, or physics-informed constraints to further enhance interpretability and accelerate the discovery pipeline.

In summary, this work establishes the feasibility of KANs as both predictive tools and interpretable models for complex materials datasets, marking a step toward transparent and data-driven materials design. Moreover, the demonstrated compositional invariance and stability of KANs-particularly in electronically complex systems-underscore their suitability for robust and physically consistent materials screening at scale.

## Supplementary Information

Supplementary Information.

## Data Availability

All scripts used for data preprocessing, feature integration, model training, KAN optimisation, and cross-validation are openly available at: https://github.com/fronzi/Kolmogorov-Arnold-Networks-Thermoelecric-Materials-Design/tree/main.
