# Supplementary material for: Interpretable machine learning for thermoelectric materials design with Kolmogorov–Arnold networks
Source: Sci Rep. 2026 Mar 19;16:14146. doi: 10.1038/s41598-026-44723-x (PMC13136493; doi:10.1038/s41598-026-44723-x)
Supplement: Supplementary file 1 — Supplementary Information. [file 41598_2026_44723_MOESM1_ESM.pdf]

Supplementary Information

Interpretable Machine Learning for Thermoelectric Materials Design with Kolmogorov–Arnold Networks

Marco Fronzi,[1,*] Michael J Ford,[2] Kamal Singh Nayal,[3] Olexandr Isayev,[3] and Catherine Stampfl[1]

[1]*School of Physics, The University of Sydney, Camperdown, Australia*

[2]*School of Mathematics and Physics, The University of Technology Sydney, Haymarket, Australia*

[3]*Department of Chemistry, Carnegie Mellon University, Pittsburgh, United States*

(Dated: February 24, 2026)

OVERVIEW

This Supplementary Information provides quantitative diagnostics supporting the robustness, generalisation behaviour, and interpretability analyses of the Kolmogorov–Arnold Network (KAN) and multilayer perceptron (MLP) models presented in the main text. All numerical values correspond to the held-out test set unless otherwise specified. The material is organised as follows:

S1. BENCHMARKING PROTOCOL FOR CONVENTIONAL MACHINE LEARNING MODELS

To provide a rigorous and fair baseline comparison, we trained and evaluated seven conventional machine learning regression models on the same 128-dimensional CrystalFormer embeddings used for the KAN and MLP models. The embedding vectors correspond to the `feature` columns of:

`train_embeddings_250epochs_embdim128_original.csv`,

generated from a 250-epoch pretrained Crystalformer encoder. All experiments were conducted with a fixed random seed (`SEED` = 42) to ensure reproducibility. When specified, a random subset of 1,000 structures was selected from the full dataset to reduce computational

cost while preserving representative sampling. The same subset was used consistently across all models and both target properties.

S1.1 Data splitting and preprocessing

For each target property, the dataset was partitioned into training and test sets using an 80/20 random split (`test_size` = 0.2). Input features were standardised using `StandardScaler`, fitted exclusively on the training set and applied to both partitions to prevent information leakage. For Seebeck coefficient regression, a sign-preserving logarithmic transformation,

$$y' = \mathrm{sign}(y)\,\log(1 + |y|), \tag{1}$$

was applied to the target variable prior to standardisation.

S1.2 Hyperparameter optimisation

Hyperparameter optimisation was performed using `RandomizedSearchCV` with the following settings:

- 25 randomly sampled configurations per model ($n_{\mathrm{iter}} = 25$),
- 5-fold cross-validation ($k = 5$),
- optimisation objective: negative root mean squared error,
- parallelised execution (`n_jobs` $= -1$).

The total number of model fits per algorithm was therefore $N_{\mathrm{fits}} = n_{\mathrm{iter}} \times k = 125$. After optimisation, the best-performing estimator was retrained on the full training partition and evaluated on the held-out test set.

S1.3 Models and search spaces

The following models were benchmarked. Search spaces were defined to span representative complexity regimes for each model class.

a. Random Forest `n_estimators`: {200, 500, 800}; `max_depth`: {None, 10, 20, 40}; `min_samples_split`: {2, 5, 10}; `max_features`: {`sqrt`, `log2`}.

b. XGBoost `n_estimators`: {300, 600, 1000}; `learning_rate`: {0.01, 0.05, 0.1}; `max_depth`: {4, 6, 8}; `subsample`: {0.7, 0.9, 1.0}; `colsample_bytree`: {0.7, 0.9, 1.0}. Early stopping with patience = 50 rounds was applied based on validation performance.

c. LightGBM and CatBoost Analogous search spaces to XGBoost, with model-specific parameters (e.g., `num_leaves` for LightGBM, `depth` for CatBoost) tuned within their recommended ranges.

d. Support Vector Regression (SVR) and Kernel Ridge Regression (KRR) `C` (SVR) / `alpha` (KRR): log-spaced grid over $[10^{-2}, 10^{3}]$; `gamma`: {`scale`, `auto`}; kernel: RBF.

e. Kolmogorov–Arnold Network (KAN) Grid size: {3, 5, 7}; regularisation coefficient λ: $\{0, 10^{-4}, 10^{-3}\}$; width configuration varied within architecture constraints; optimiser: LBFGS.

f. Multi-Layer Perceptron (MLP) Hidden layer sizes: {(128, 64), (256, 128), (256, 256)}; activation: {ReLU, GELU}; learning rate: $\{10^{-3}, 5\times10^{-4}\}$; weight decay: $\{0, 10^{-4}, 10^{-3}\}$; batch size: {64, 128}; training epochs: up to 300 with early stopping (patience = 30).

g. TabPFN-2.5 The default regression checkpoint;
(`tabpfn-v2.5-regressor-v2.5_default.ckpt`) was used without fine-tuning, following the inference protocol recommended by the developers[1]. TabPFN operates via in-context learning and approximates Bayesian posterior inference over regression problems in a single forward pass without dataset-specific gradient optimisation; no conventional hyperparameter tuning was therefore performed.

S1.4 Evaluation metrics

Predictive performance on the held-out test set was quantified using the coefficient of determination (R^2), mean absolute error (MAE), root mean squared error (RMSE), and

mean squared error (MSE):

$$R^2 = 1 - \frac{\sum_i (y_i - \hat{y}_i)^2}{\sum_i (y_i - \bar{y})^2}, \quad (2)$$

$$\text{MAE} = \frac{1}{N} \sum_i |y_i - \hat{y}_i|, \quad (3)$$

$$\text{RMSE} = \sqrt{\frac{1}{N} \sum_i (y_i - \hat{y}_i)^2}, \quad (4)$$

$$\text{MSE} = \frac{1}{N} \sum_i (y_i - \hat{y}_i)^2. \quad (5)$$

Results for each target property were stored separately and subsequently combined into the benchmarking tables in Section S2.

S2. BENCHMARK PERFORMANCE TABLES

Tables S1 and S2 report test-set performance for all benchmarked models on band gap and Seebeck coefficient prediction, respectively, sorted by descending R^2.

TABLE S1. Test-set performance for band gap prediction, sorted by descending R^2. Errors are in eV.

Model	R^2	MAE (eV)	RMSE (eV)	MSE (eV^2)
SVR	0.970	0.174	0.306	0.093
CatBoost	0.968	0.179	0.316	0.100
KernelRidge	0.968	0.190	0.318	0.101
XGBoost	0.966	0.187	0.328	0.107
LightGBM	0.964	0.199	0.339	0.115
RandomForest	0.961	0.199	0.353	0.125
MLP	0.957	0.243	0.367	0.134

Tables S3 and S4 report five-fold cross-validation performance of TabPFN-2.5, for band gap and Seebeck coefficient prediction respectively. TabPFN-2.5 is a transformer-based foundation model for tabular data that performs in-context learning by conditioning on the entire training dataset during inference[1]. Pre-trained on a large distribution of synthetic tabular tasks, it approximates Bayesian posterior inference over regression problems in a

TABLE S2. Test-set performance for Seebeck coefficient (S_n) prediction, sorted by descending R^2. Errors are in μV/K.

Model	R^2	MAE (μV/K)	RMSE (μV/K)	MSE (μV/K^2)
LightGBM	0.934	51.7	86.7	7,517.7
KernelRidge	0.927	59.4	91.2	8,319.5
CatBoost	0.925	53.7	92.2	8,503.1
XGBoost	0.915	52.2	98.4	9,691.2
SVR	0.914	61.2	98.6	9,715.4
MLP	0.911	64.2	100.2	10,045.9
RandomForest	0.895	56.3	109.1	11,896.6

single forward pass without dataset-specific gradient optimisation. The default regression checkpoint was used in this study.

TABLE S3. Five-fold cross-validation performance of TabPFN-2.5 (default regression checkpoint) for band gap prediction. Errors are in eV. The final row reports mean ± standard deviation across folds.

Fold	R^2	MAE (eV)	RMSE (eV)	MSE (eV^2)
1	0.9764	0.152	0.295	0.087
2	0.9757	0.169	0.279	0.078
3	0.9558	0.179	0.385	0.149
4	0.9591	0.158	0.375	0.141
5	0.9809	0.142	0.259	0.067
Mean ± Std	0.970 ± 0.012	0.160 ± 0.014	0.319 ± 0.055	0.104 ± 0.034

TabPFN-2.5 demonstrates strong predictive capability for band gap estimation ($R^2 = 0.970 \pm 0.012$), with low absolute and quadratic errors across folds. Performance on the Seebeck coefficient remains robust ($R^2 = 0.892 \pm 0.008$), though with slightly higher dispersion, consistent with the intrinsically broader distribution of thermoelectric response values. The limited variance across folds indicates stable generalisation on the selected subset.

TABLE S4. Five-fold cross-validation performance of TabPFN-2.5 (default regression checkpoint) for Seebeck coefficient (S_n) prediction. The final row reports mean ± standard deviation across folds.

Fold	R^2	MAE	RMSE	MSE
1	0.8911	0.349	0.720	0.518
2	0.8843	0.371	0.707	0.500
3	0.9059	0.348	0.664	0.441
4	0.8886	0.418	0.713	0.508
5	0.8920	0.364	0.706	0.499
Mean ± Std	0.892 ± 0.008	0.370 ± 0.026	0.702 ± 0.020	0.493 ± 0.028

S3. ELEMENT-RESOLVED ERROR TRENDS

Table S5 reports group-averaged MAE values corresponding to the periodic-table maps shown in the main text. Values illustrate systematic spatial trends rather than exact numerical extraction.

TABLE S5. Group-averaged MAE values for KAN and MLP across representative chemical regions. Values are approximate, reflecting spatial trends rather than precise per-element extraction.

Chemical region	Property	KAN MAE	MLP MAE	Qualitative trend
Light p-block (G13–17)	Band gap (eV)	∼0.20–0.30	∼0.30–0.45	MLP shows local spikes
Light p-block (G13–17)	Seebeck (μV/K)	∼40–60	∼60–90	Broader MLP variance
Late transition metals	Band gap (eV)	∼0.25–0.40	∼0.35–0.55	Sharper MLP contrast
Late transition metals	Seebeck (μV/K)	∼70–100	∼80–130	More localised peaks in MLP
Alkali / alkaline-earth	Band gap (eV)	∼0.10–0.20	∼0.10–0.25	Comparable performance

KAN errors are more spatially uniform across chemical families, whereas MLP displays sharper element-dependent variability, particularly in transition-metal and p-block regions.

S4. COMPOSITIONAL ENTROPY AND COMPLEXITY ANALYSIS

We analysed the percentage MAE as a function of Shannon compositional entropy, $S_{\text{chem}} = -\sum_i x_i \ln x_i$, where x_i denotes the fractional atomic concentration of species i. Table S6 reports approximate MAE trends across entropy bins, and Table S7 reports the corresponding Spearman rank correlation coefficients.

TABLE S6. Approximate MAE trends across Shannon compositional entropy bins for KAN and MLP models.

Entropy range	KAN MAE (relative)	MLP MAE (relative)
Low ($S_{\text{chem}} < 0.5$)	Low	Low–Moderate
Intermediate ($0.8 < S_{\text{chem}} < 1.2$)	Moderate peak	Moderate
High ($S_{\text{chem}} > 1.8$)	Low–Moderate	Moderate

TABLE S7. Spearman rank correlation coefficients (ρ) between percentage MAE and Shannon compositional entropy, for KAN and MLP on both target properties.

Target	KAN ρ	MLP ρ
Band gap	+0.012	+0.009
Seebeck (S_n)	−0.006	+0.015

All Spearman coefficients satisfy $|\rho| \leq 0.015$, confirming the absence of a statistically significant monotonic relationship between compositional entropy and prediction error for either architecture. Both architectures remain stable across compositional complexity regimes.

S5. ROBUSTNESS DIAGNOSTICS AND PHYSICAL CONSISTENCY

Table S8 reports key robustness diagnostics on the held-out test set, including the number of physically implausible predictions and the tail percentiles of the absolute error distribution.

KAN exhibits zero Seebeck sign inversions, whereas MLP produces 35 sign-inconsistent predictions. Both models occasionally generate negative band-gap values, with MLP doing so more frequently (371 vs. 308 cases). KAN's spline continuity suppresses physically inconsistent extrapolations.

TABLE S8. Robustness diagnostics on the held-out test set. Sign inversions denote cases where the predicted Seebeck carrier type is reversed relative to the true value. Negative predictions denote cases of unphysical negative band-gap values. Percentile errors are in eV (band gap) and μV/K (Seebeck).

Property	Model	Sign inv.	Neg. pred.	95% err.	99% err.
Band gap	KAN	–	308	0.52	0.99
Band gap	MLP	–	371	0.54	1.02
Seebeck	KAN	102	–	122.0	289.9
Seebeck	MLP	329	–	186.3	505.5

S6. DESCRIPTOR–TARGET CORRELATION ANALYSIS

Table S9 reports Pearson (r) and Spearman (ρ) correlation coefficients between the three CrystalFormer descriptors with the highest KAN attribution scores (x_{39}, x_{68}, x_{83}) and each target property. The maximum observed $|r|$ across all 128 features is reported in the final row.

TABLE S9. Pearson (r) and Spearman (ρ) correlation coefficients between the three highest-attribution CrystalFormer descriptors and each target property. The maximum observed $|r|$ across all 128 features is reported in the final row.

Feature	r (gap)	ρ (gap)	r (S_n)	ρ (S_n)	r (S_p)	ρ (S_p)
x_{39}	0.154	0.131	0.143	0.118	0.172	0.149
x_{68}	0.147	0.122	0.095	0.081	0.133	0.109
x_{83}	0.071	0.082	0.112	0.068	0.160	0.127
Max $|r|$ (all 128) < 0.20 across all features and all target properties						

No descriptor exhibits strong linear correlation with any target property ($|r| < 0.20$ throughout). Predictive performance therefore arises from nonlinear multivariate interactions.

S7. KAN ATTRIBUTION AND SPARSITY DIAGNOSTICS

S7.1 Test-set performance

$$R^2 = 0.947, \tag{6}$$

$$\text{RMSE} = 0.376 \text{ eV}, \tag{7}$$

$$\text{MAE} = 0.207 \text{ eV}. \tag{8}$$

Median absolute error: 0.132 eV;   99th percentile error: 1.652 eV.

S7.2 Feature attribution at the input layer (L0)

Top 10 features account for 11.8% of attribution mass. 67 features are required to reach 50% of total attribution.

- Gini coefficient: 0.128
- Normalised entropy: 0.984

Attribution is therefore broadly distributed across the 128 input descriptors.

S7.3 Hidden node attribution at the hidden layer (L1)

The top 3 hidden nodes account for 41.6% of total node attribution mass. Attribution becomes significantly more concentrated at the hidden layer, indicating emergent functional bottlenecks.

S7.4 Edge-level attribution and pruning strategy

The strongest edges at L0 primarily terminate on the dominant hidden nodes identified in S7.3, confirming structural consistency between node-level and edge-level attribution. Edge-level sparsification after pruning is present but moderate: the network retains a broadly connected structure at L0, while L1 exhibits selective concentration.

The L_n scores provide a quantitative basis for model compression:

- Retain hidden nodes until a cumulative attribution threshold is reached.
- Within retained nodes, prune low-scoring edges.

Given the diffuse feature-level attribution, aggressive input pruning is not recommended without retraining.

---

* marco.fronzi@sydney.edu.au

[1] L. Grinsztajn, K. Fl oge, O. Key, F. Birkel, P. Jund, B. Roof, B. Jger, D. Safaric, S. Alessi, A. Hayler, M. Manium, R. Yu, F. Jablonski, S. B. Hoo, A. Garg, J. Robertson, M. Bhler, V. Moroshan, L. Purucker, C. Cornu, L. C. Wehrhahn, A. Bonetto, B. Schlkopf, S. Gambhir, N. Hollmann and F. Hutter, *TabPFN-2.5: Advancing the State of the Art in Tabular Foundation Models*, 2025, https://arxiv.org/abs/2511.08667.
